# Identification and expression analysis of nuclear factor Y transcription factor genes under drought, cold and Eldana infestation in sugarcane (Saccharum spp. hybrid)

**DOI:** 10.1007/s13258-024-01529-3

**Published:** 2024-06-14

**Authors:** Jancke le Roux, Robyn Jacob, Riëtte Fischer, Christell van der Vyver

**Affiliations:** 1 Institute for Plant Biotechnology, Department of Genetics, University of Stellenbosch, Stellenbosch, 7602 South Africa; 2 South African Sugarcane Research Institute (SASRI), KwaZulu-Natal, P/Bag X02, Mount Edgecombe, Durban, 4300 South Africa

**Keywords:** NF-Y transcription factors, Abiotic and biotic stress responses, Sugarcane, Eldana

## Abstract

**Background:**

The Nuclear Factor Y (NF-Y) transcription factor (TF) gene family plays a crucial role in plant development and response to stress. Limited information is available on this gene family in sugarcane.

**Objectives:**

To identify sugarcane NF-Y genes through bioinformatic analysis and phylogenetic association and investigate the expression of these genes in response to abiotic and biotic stress.

**Methods:**

Sugarcane NF-Y genes were identified using comparative genomics from functionally annotated Poaceae and Arabidopsis species. Quantitative PCR and transcriptome analysis assigned preliminary functional roles to these genes in response to water deficit, cold and African sugarcane borer (*Eldana saccharina*) infestation.

**Results:**

We identify 21 NF-Y genes in sugarcane. Phylogenetic analysis revealed three main branches representing the subunits with potential discrepancies present in the assignment of numerical names of some NF-Y putative orthologs across the different species. Gene expression analysis indicated that three genes, ShNF-YA1, A3 and B3 were upregulated and two genes, NF-YA4 and A7 were downregulated, while three genes were upregulated, ShNF-YB2, B3 and C4, in the plants exposed to water deficit and cold stress, respectively. Functional involvement of NF-Y genes in the biotic stress response were also detected where three genes, ShNF-YA6, A3 and A7 were downregulated in the early resistant (cv. N33) response to Eldana infestation whilst only ShNF-YA6 was downregulated in the susceptible (cv. N11) early response.

**Conclusions:**

Our research findings establish a foundation for investigating the function of ShNF-Ys and offer candidate genes for stress-resistant breeding and improvement in sugarcane.

**Supplementary Information:**

The online version contains supplementary material available at 10.1007/s13258-024-01529-3.

## Introduction

Transcription factors (TFs) are proteins with DNA-binding domains which can recognize and bind specific DNA elements in promoters and enhancers to control the transcription of DNA into RNA. Transcription factors therefore initiate and regulate the transcription of target genes (Franco-Zorrilla et al. 2014). They are organized into multigene families and are involved in plant developmental control and stress responses (Shiu et al. 2005).

The Nuclear Factor Y (NF-Y) transcription factor gene family specifically binds to the CCAAT-box in eukaryotic promoters to regulate the expression of their target genes. These TFs are therefore also known as CCAAT Binding Factors (CBF) or Heme Activator Proteins (HAP). NF-Y is composed of a complex made up by the three subunits NF-YA, NF-YB and NF-YC (Nardini et al. 2013). Individual NF-Y subunits cannot control transcription independently but must function in heterodimers or heterotrimers. Interactions between the subunits are facilitated by a histone fold domain present on the NF-YB and NF-YC subunits which ensure stable interaction with the NF-YA subunit (Baxevanis et al. 1995; Zemzoumi et al. 1999). The NF-YA subunit has two α-helix structure domains, namely a 20 amino acid N-terminal conserved α-helix A1 domain, responsible for interaction with NF-YB and NF-YC, and a 21 amino acid C-terminal α-helix A2 domain which binds with the CCAAT element. These interactions between the subunits mean that many possible NF-Y complexes can form in flexible combinations.

In plants, each NF-Y subunit family is encoded by multiple genes. In *Arabidopsis*, updated analysis of the NF-Y TF gene family identified 30 genes, 10 each coding for the three subunit families (Siefers et al. 2009; Petroni et al. 2012; Zhao et al. 2017). Within the Poaceae grass family the number of NF-YA, NF-YB and NF-YC genes vary, in *Brachypodium distachyon* 7, 17 and 12 (Cao et al. 2011), in rice the updated number of 11, 11 and 12 (Thirumurugan et al. 2008; Yang et al. 2017), in maize 14, 18 and 18 (Zhang et al. 2016), respectively, and in wheat 10, 11 and 14 (Stephenson et al. 2007) or even as many as 18, 34 and 28 if the A, B and D genomes are combined (Qu et al. 2015), were identified.

It is well documented that members of the NF-Y gene families play important roles in developmental control, such as flowering (Kumimoto et al. 2008; Hwang et al. 2016; reviewed Petroni et al. 2012; Stephenson et al. 2010), seed development (Lee et al. 2003; Lian et al. 2018), nutrient control (Pant et al. 2009; Qu et al. 2015), hormone control (Zhang et al. 2017), root nodule/microbe interactions (reviewed Zanetti et al. 2016) and photosynthesis (Wang et al. 2018). These TFs are also known to enhance abiotic stress tolerance in terms of drought, salt, and cold (Han et al. 2013; Ma et al. 2015; Lian et al. 2018; Wang et al. 2018). For example, the *CsNFYA5* gene from citrus, when overexpressed in tobacco, resulted in enhanced drought tolerance in the transgenic plants due to reduced reactive oxygen species production and increased photosynthesis and under normal conditions increased plant growth due to an increased photosynthetic rate (Pereira et al. 2018). Possible roles in biotic stress are less well documented. By analyzing gene expression with qPCR and available microarray data, Ren et al. (2016), suggested that NF-Y genes in grapevine might participate in responses to biotic stresses.

Even though the NF-Y gene family has been studied and reviewed in many plant species, little is known about NF-Y genes in sugarcane. A recent genome-wide association study identified several NF-Y genes (Clarancia et al. 2023) in a monoploid reference genome of sugarcane (Garsmeur et al. 2018), but to our knowledge, no sugarcane NF-Y genes have been functionally annotated. Sugarcane, belonging to the genus *Saccharum* in the grass family Poaceae (Moore et al. 2013), is an important crop cultivated worldwide primarily for sucrose production and as a biomass resource for biofuel production (Rabelo et al. 2020). Commercial varieties have ploidy levels that ranges from 5x to 16x and chromosome numbers between 2n = 99 and 130 (Butterfield et al. 2001; Manners et al. 2004), with a genome size between 3.36 and 12.64 Gb (Souza et al. 2011; Thirugnanasambandam et al. 2018; Trujillo-Montenegro et al. 2021). Each commercial genotype (*Saccharum* species hybrid) has a random mixture of the chromosome originating likely from two polypoid progenitor species, *S. officinarum* and *S. spontaneum*. This genome complexity and high gene copy number have delayed and limited our understanding and annotation of the sugarcane genome (Thirugnanasambandam et al. 2018). In the current study, we identify several sugarcane NF-Y genes through bioinformatic analysis and investigate their phylogenetic associated with putative NF-Y orthologs in other Poaceae crop species with potentially similar functions. Additionally, we investigated the expression patterns of these NF-Y genes in sugarcane in response to water deficit and, for the first time, to cold stress and African sugarcane borer (*Eldana saccharina*) infestation using available transcriptome data.

## Materials and methods

### Database searches for NF-Y gene family members

To collect all the members of the sugarcane NF-Y gene family, we identified the conserved amino acid core regions in the NF-YA, NF-YB and NF-YC subunits of the *A. thaliana* gene family from the Arabidopsis Information Resource (TAIR) database (https://www.arabidopsis.org/), and from NF-Y genes in *Oryza sativa*, *Zea ma*ys and *Sorghum bicolor* available at the Phytozome 12 database (https://phytozome.jgi.doe.gov/pz/portal.html). These conserved regions were used as queries to search the Sugarcane Genome Hub (https://sugarcane-genome.cirad.fr/) and the references of *Saccharum* listed on the National Centre for Biotechnology Information (NCBI) database (https://www.ncbi.nlm.nih.gov/). Only the longest sequence was retained if several results appeared to be from the same gene. The sugarcane *NF-Y* genes were assigned names as presented on the Sugarcane Genome Hub or, alternatively, in reference to the highest nucleotide-nucleotide NCBI BLAST hit with the NF-Y genes in other Poaceae species. Priority was given to hits in *S. bicolor*, then *Z. mays*, then *O. sativa*, and finally other grasses.

### Analysis of NF-Y sequences

To obtain information regarding coding sequences, exon-intron structure and predicted amino acid sequences of the identified sugarcane NF-Ys, data was obtained from the *S. bicolor* v3.1.2 genomic resource https://phytozome.jgi.doe.gov/pz/ portal.html#!info? alias = Org_Sbicolor) hosted on the Phytozome database. In addition, seemly partial amino acid NF-Y sequences obtained from the database searches were extended by a bait-and-build pipeline like that described in Evans and Joshi (2016). Briefly, this uses the MIRABAIT component of the MIRA assembly software (Chevreux et al. 2004) to extract short sequencing reads from *Saccharum* species hybrid cultivar N33 RNA-seq data by mapping the reads to the partial transcript sequence. The baited reads are then assembled into an extended contig using SPAdes (Bankevich et al. 2012).

To find available genome sequences, TBLASTN was used to Blast the sugarcane cultivar SP80-3280 whole-genome shotgun contigs (WGS) database (Accession: PRJNA431722) of NCBI. To predict the molecular weight and isoelectric point (pi) of the inferred amino acid sequences the PROTPARAM software tool (https://web.expasy.org/cgi-bin/compute_pi/pi_tool) on the Expert Protein Analysis System (ExPASy) proteomics database (http://www.expasy.ch/) was used.

### Multiple sequence alignments and phylogenetic assessment

Multiple sequence alignments were created with the identified full length protein sequences of the sugarcane NF-YA, NF-YB and NF-YC gene family members and the corresponding protein sequences from other Poaceae grass families and *A. thaliana* using the CLC Sequence viewer 8 software (https://clc-sequence-viewer.software.informer.com/8.0/).T The conserved domains were represented visually using WebLogo (https://weblogo.berkeley.edu/logo.cgi) for each subunit. Subsequently, unrooted phylogenetic trees were created using the MEGA11.0.10 software (https://www.megasoftware.net/) with the neighbor-joining method and bootstrap values from 1000 replicates at each branch.

### Plant material and stress treatments

Sugarcane plantlets (*Saccharum* species hybrid cv. NCo310) were multiplied in vitro on semi-solid medium containing 4.4 g /L MS basal salts (Murashige and Skoog 1962), 2% (w/v) sucrose, and 0.22 g/L gelrite (pH 5.8). In vitro plantlets, 5–10 cm high with roots, were hardened off in the glasshouse in 20 cm diameter pots in potting mixture consisting of potting soil, vermiculite, and sand in a 2:1:1 ratio and fertilized every two weeks with 3 g/l Hygrotech (Hygrotech Properties (PTY) LTD, South Africa) and 2.5 g/l CaNO_3_. The glass house settings included daily watering via a sprinkler system, natural light and temperatures at 26 ± 2˚C. Plants were allowed to grow for five months after which the plants were exposed to 30 days of water deficit stress by withholding water. Plant tissue, the top visible dewlap (TVD) leaf from each plant, four plants per treatment, was harvested prior to the stress induction and at the end of the stress period (day 30 without water [ww]). In addition, a second set of in vitro sugarcane plantlets were exposed to cold stress at 4 °C for 48 h in vitro, after which leaf tissue was harvested from both stressed and unstressed, kept at 26 °C, plantlets. Four biological replicates were collected for each treatment condition. Samples were flash frozen in liquid nitrogen and stored at -80˚C until further analysis.

### Quantitative real-time PCR analysis (qPCR)

Total RNA was isolated from the leaf tissue (100 mg) from stressed and control plants using the Maxwell 16® LEV Plant RNA kit (Promega, Madison, WI, USA) according to the manufacturer’s specifications. RNA was treated with DNase I and added to the Maxwell cartridge for processing. Complementary DNA (cDNA) was synthesized from 1 µg total RNA using the RevertAid First Strand cDNA Synthesis kit (Thermo Scientific, Waltham, USA) following the manufacturer’s instructions.

Transcript levels for the sugarcane ShNF-Y family members were quantified by qPCR using a Quant Studio 3 Real-Time PCR System and the PowerUP SYBR Green Master mix (Thermo Fisher Scientific). Gene-specific primers pairs were designed for amplicons ± 120 bp long (Supplementary Table S1) and conventional PCR was performed to ensure a single amplicon was generated for each gene (Supplementary Fig. S1). Three reference genes were tested as internal controls, *glyceraldehyde-3-phosphate dehydrogenase (GAPDH;* CA254672), *ubiquitin (UBQ1;* CA094944) and *actin (ACT;* CA148161*)* (De Andrade et al. 2017). A dilution range of cDNA samples were subjected to qPCR to collect Ct values. Data were obtained from a pool of four biological replicates, each validated with three technical repeats. The expression values of the genes were calculated using the QuantStudio 3 Design and Analysis Software v1.3.1 software, and the Ct values were exported to Microsoft Office Excel v2013, for further calculations as described by Livak and Schmittgen (2001).

### Statistical analysis

Statistical analysis was carried out with the XLSTAT v19.1 software (Addinsoft, Paris, France). Error bars are shown as standard error to the mean values calculated from biological replicates (*n* = 4). Data was tested for normal distribution with the Shapiro-Wilk normality test. Statistically significant differences were assessed based on the analysis of variance (ANOVA) for data showing normal distribution and subjected to the Dunnett two-sided analysis of the differences from the control. Data that failed the normality test was further tested through a non-parametric test (Kruskal-Wallis test). P-values of ≤ 0.05 were considered statistically significantly different from control samples and displayed with an asterisk in the different data graphs.

### Transcriptome analysis

Novel sugarcane NF-Y genes were identified from a *de novo* assembled transcriptome derived from the RNA pool of two *Saccharum* hybrid species cultivars (N33 and N11). The twenty-four sequenced libraries were constructed from the stalk tissue of three biological replicates of each cultivar challenged with the stalk borer, *E. saccharina* (eldana), at two time-points and three biological replicates of unchallenged controls, at the same time-points. The transcriptome was annotated using the Eukaryotic Non-Model Transcriptome Annotation Pipeline (EnTAP) (Hart et al. 2020) incorporating annotations from NCBI non-redundant database (release 238.0), SwissProt (release 2020_03), Plant Refseq (release 201) and the Arabidopsis proteome (release 20,101,214).

The set of *ShNF-Y* gene sequences derived from database searches were appended to the transcriptome for the purposes of investigating the abundance of reads mapping to this set. Transcript abundances (transcript per million; TPM) in the eldana-challenged and unchallenged eldana resistant (N33) and susceptible (N11) cultivars at two time points (d1: 1 day after challenge; d2: 3 days after challenge) were determined using Kallisto version 0.46.0 (Bray et al. 2016). The kallisto abundance files were imported into R (version 4.0.2) using the tximport package (Soneson et al. 2016), and transcript level abundance estimates were summed for a gene-level analysis. Genes with no/low read support were removed from the analysis. Sleuth 0.30.1 (Pimentel et al. 2017) was used in R version 4.1.2 to perform differential analysis using the transcript-level abundance estimates output by kallisto.

## Results and discussion

This study employed comparative genomics to identify sugarcane NF-Y transcription factor genes using data from functionally annotated sorghum, maize, rice and Arabidopsis species. Based on qPCR and transcriptome analysis, preliminary functional roles were assigned to the NF-Y genes in sugarcane, focusing on their involvement in the abiotic and biotic stress response.

### Multiple alignments and in silico analysis of ShNF-Y

**Fig. 1:**
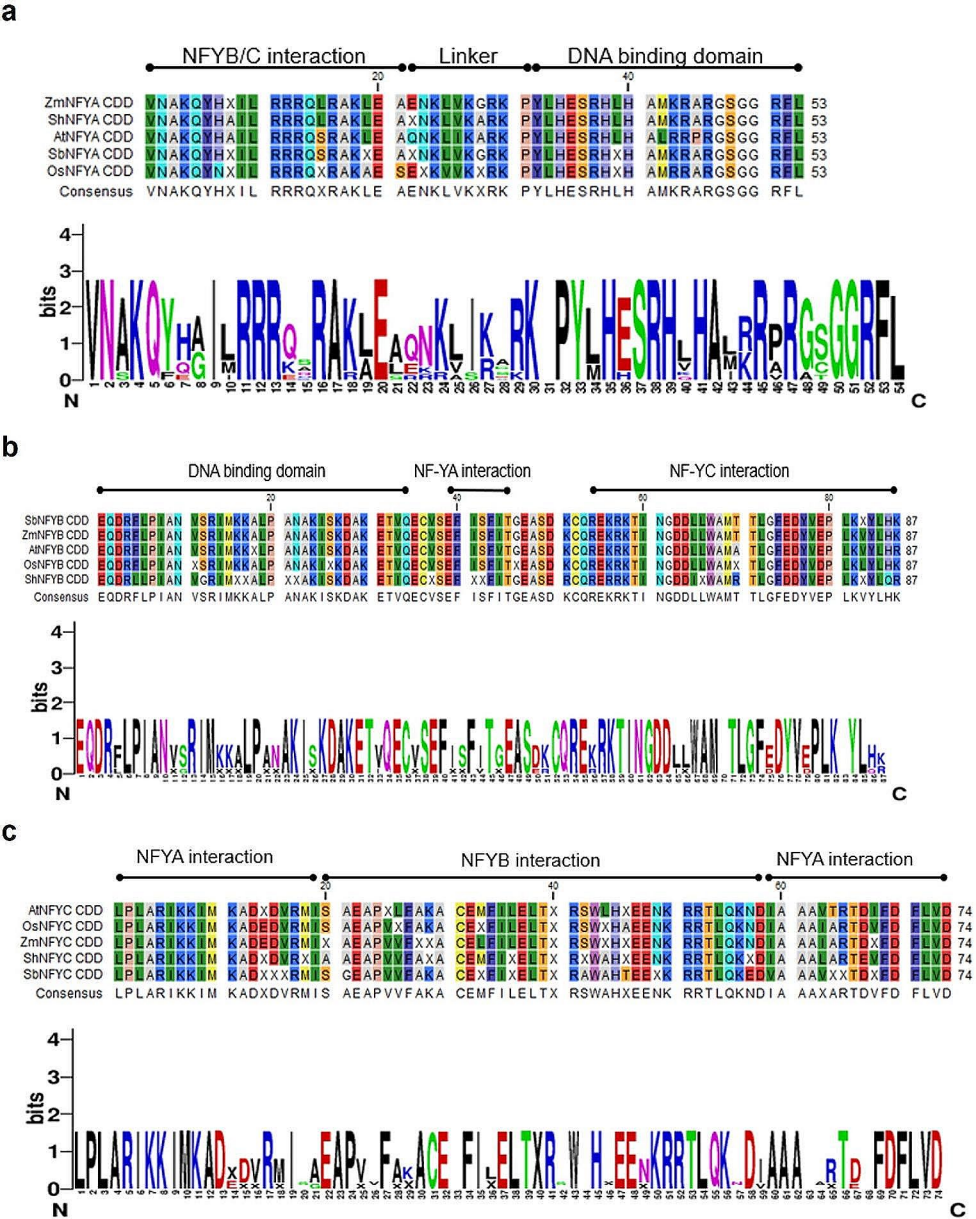
Multiple alignment of the conserved region identified in the **(a)** NF-YA, **(b)** NF-YB and **(c)** NF-YC subunit gene families across multiple plant species. Multiple alignments were performed with the CLC sequence viewer software and the most prevalent amino acid sequence displayed by WebLogo (https://weblogo.berkeley.edu/logo.cgi). Plant species include *A. thaliana* (At), *Z. mays* (Zm), *O. sativa* (Os), *S. bicolor* (Sb) and *Saccharum* hybrid (Sh)

Multiple protein sequence alignments indicated the conserved amino acid region within each of the three NF-Y subunit families, with the interaction domains highlighted (Fig. 1). Across plant species, ShNF-Y proteins have clear regions of homology flanked by largely non-conserved sequences in the C-terminus region. These conserved regions, approximately 62-amino acids in length, are necessary to facilitate protein subunit heterodimerization, heterotrimerization, and DNA binding at the CCAAT regions in the target gene promoters (Romier et al. 2003; Cao et al. 2011; Petroni et al. 2012). The conserved regions were used as queries in all further database searches to identify the *NF-Y* gene family members in sugarcane.

BLAST searches using the ∼62 amino acid conserved regions for each NF-Y subunit as query sequences resulted in the identification of 21 NF-Y proteins in sugarcane, encoded by nine NF-YA, seven NF-YB and five NF-YC genes (Table 1). This number of identified NF-Y genes is similar to the number of NF-YA but less than the number of NF-YB and C genes identified in the mosaic monoploid sugarcane reference genome, which was assigned numerical numbers according to their physical chromosome positions, as recently reported by Clarancia et al. (2023). Of the 18 NF-YB genes described by Clarancia et al. (2023), 10 genes, (*NF-YB1-B8*, *B12* and *B13*) had identical coding sequences (CDS) and protein lengths (312 bp and 103 amino acids), consensus motifs, and phylogenetic branching (100), but showed some variability in the ORF length. In the current study, these were likely considered similar genes. However, the nucleotide and amino acid sequences of these reported NF-YB genes were not released, making direct sequence comparisons impossible. Gene sequences will first have to be re-mined from the database reference genome based on the reported estimated chromosome positions before such alignments can be made. Furthermore, we were unable to find the large number of NF-YC genes (24) reported by Clarancia et al. (2023). Known data indicate NF-YC gene numbers vary between 12 and 18 in Poaceae species (Stephenson et al. 2007; Thirumurugan et al. 2008; Cao et al. 2011; Yang et al. 2017; Zhang et al. 2016), which is more than the five NF-YC genes we were able to find. Renewed attempts will be required to identify these additional putative NF-YC genes.

**Table 1:** Assigned names for identified sugarcane *NF-Y* genes based on BLAST hits on NCBI, the Sugarcane Hub and transcriptome (Trinity accessions)

Assigned gene name	Sugarcane Hub accession number	Trinity accession number	NCBI best BLAST hit	Query cover	% Identity
*ShNF-YA1*		DN222879	*SbNFY-A1* (XM_021448471.1)	100%	95.91%
*ShNF-YA2*	Sh_210J01		*SbNF-YA10* (XM_002461436.2)	86%	87.31%
*ShNF-YA3*	Sh_215C15	DN226448	*SbNF-YA3* (XM_021453812.1) *ZmNF-YA3* (NM_001153839.2)	99% 62%	94.89% 92.27%
*ShNF-YA4*		DN232303	*SbNFY-A4* (XM_021449293.1)	100%	96.24%
*ShNF-YA5*	Sh_142B14	DN222855	*SbNFY-A5* (XM-021458675.1) *SbNF-YA5* (XM_021458677.1)	100% 97%	95.87% 93.25%
*ShNF-YA6*	Sh_239I08	DN218789	*SbNFY-A7* (XM_002465725.2)	100%	94.81% 94.95%
*ShNF-YA7*	Sh_254O14	DN230864	*SbNFY-A7* (XM_002443504.2)	100% 95%	97.05% 93.16%
*ShNF-YA10*	Sh_232I01	DN233450	*SbNFY-A10* (XM_021445712.1)	100%	91.84%
*ShNF-YA13*	Sh_022O20		*ZmNFY-A13* (KM_655751.1)	75%	91.68%
*ShNF-YB1*	Sh_241A20		*SbNF-YB1* (XM_002452581.2)	82%	90.53%
*ShNF-YB2*	Sh_2441P06		*SbNF-YB2* (KF533110.1)	94%	99.15%
*ShNF-YB3*		DN218159	*SbNF-YB3* (XP_002458721.1)	90%	99.32%
*ShNF-YB4*	Sh_235I12	DN236822	*SbNF-YB4* (XM_021446908.1)	82% 98%	96.01% 96.10%
*ShNF-YB5*	Sh_243O13_p000150		*SvNF-YB4* (XM_034741816.1)	98%	82.34%
*ShNF-YB6*	Sh_241A20_p000080		*SbNF-YB2* (XM_002452582.2)	100%	90.44%
*ShNF-YB10*		DN237620	*SbNF-YB10* (XM_002463118.2)	98%	97.94%
*ShNF-YC2*		DN238653	*ZmNF-YC2* (NM_001156154.2)	100%	92.58%
*ShNF-YC3*	Sh_215M05_t000070	DN192942_206940	*SbNF-YC3* (XM_021465359.1)	100% 98%	88.64% 89.03%
*ShNF-YC4*		DN220412	*SbNF-YC4* (XM_002443915.2)	100%	94.95%
*ShNF-YC6*	Sh_202L17 Sh_201J12		*SbNF-YC6* (XM_002460379.2)	100%	99.01% 98.59%
*ShNF-YC9*	Sh_201O15		*ZmNF-YC9* (NM_001151092.2)	100%	89.29%

In the current study, each gene was named with a two-letter code corresponding to *Saccharum* hybrid (Sh), followed by NF-Y and the subunit designation (A, B, or C), and finally a number. The best nucleotide BLAST results from NCBI, the Sugarcane Genome Hub (https://sugarcanegenome.cirad.fr/) and the Phytozome database, based on e-values, query coverage and percentage identity, were used to assign NF-Y names for each identified non-annotated putative sugarcane NF-Y gene sequence. When naming the genes, priority was given in the following species order, first, sugarcane (*Saccharum* sp.), then sorghum (*S. bicolor*), followed by other grass species such as maize (*Z. mays*) and rice (*O. sativa*), when there were no BLAST hits in the listed species (Table 1). Additionally, NF-Y query sequences were used in homology searches of the available sugarcane transcriptome to identify novel/non-annotated/partial ShNF-Y transcripts (Table 1). The polyploid nature of sugarcane also means that there likely exist multiple copies of each NF-Y gene in the sugarcane genome. For instance, the Sugarcane Genome Hub database lists four ShNF-YB4 entries (Sh_235I12_p000020, Sh_213G15_p0000030, Sh_243O13_p000160 and Sh_243O13_p000150) of which the first two share the exact nucleotide sequence except for three base pairs. The third and fourth accessions are likely variations of the same gene (named ShNF-YB5 in this study), based on multiple sequence alignments with sorghum, maize, and rice orthologues. The Sugarcane Genome Hub also lists two ShNF-YA5 entries, but these display a 48 amino acid difference (Sh_142B14_t000070 and Sh_022O20_t000060) and was therefore considered two different genes. They were named ShNF-YA3 and ShNF-YA13 in this study, based on the top NCBI BLAST hits against sorghum and maize. Similarly, two ShNF-YA7 entries are listed with an 84 amino acid difference (Sh_215C15_t000060 and Sh_245O14_t0000040), of which the first was named ShNF-YA3 and the second was named ShNF-YA7 in this study, based on the top NCBI BLAST hits against maize and sorghum. Furthermore, five ShNF-YC2 entries are listed (Sh_124M05_t000070, Sh_202L17_t000080, Sh_201J12_contig-1_t000050, Sh_2117O23_t000010 and Sh_215M05_t000100). The first was named ShNF-YC3, the second and third were renamed ShNF-YC6 and are likely variations of the same gene based on comparative genomic analysis with other Poaceae species. The fourth and fifth genes were found to encode F-box proteins and were excluded from this analysis. F-box proteins are a multigene family that forms one of three types of E3 ubiquitin ligases that provide substrate specificity in the cellular plant responses, in which target proteins are ubiquitinated for controlled protein degradation and can therefore not be considered part of the NF-Y TF gene family (Xu et al. 2009).

The identified genes were analyzed in terms of nucleotide length, protein length and molecular weight (Table 2). The *ShNF-YA10* gene was the longest, coding for a protein consisting of 338 amino acids (aa), while *NF-YB10* encoded for the shortest protein consisting of 131 amino acids. The nucleotide and amino acid sequences for the ShNF-Y genes are listed in the Supplementary data file S8.

**Table 2:** List of sugarcane *NF-Y* transcription factor genes. MW = molecular weight in kilo Dalton; PI = isoelectric point. The nucleotide and amino acid sequences for these genes are shown in Supplementary data S8

Name	CDS length (bp)	Protein length (aa)	MW (kDa)	PI
*ShNF-YA1*	804	268	28.2	9.42
*ShNF-YA2*	516	172	19.3	11.77
*ShNF-YA3*	897	299	32.6	8.21
*ShNF-YA4*	915	305	33.3	9.73
*ShNF-YA5*	915	305	32.9	11.18
*ShNF-YA6*	726	242	26.3	8.98
*ShNF-YA7*	645	215	23.4	7.97
*ShNF-YA10*	1164	338	36.5	9.24
*ShNF-YA13*	771	257	28.6	10.58
*ShNF-YB1*	414	138	14.9	9.28
*ShNF-YB2*	543	181	19.0	6.31
*ShNF-YB3*	492	164	17.8	5.82
*ShNF-YB4*	486	162	18.0	5.96
*ShNF-YB5*	501	167	18.4	6.52
*ShNF-YB6*	783	261	27.7	6.4
*ShNF-YB10*	393	131	14.2	5.07
*ShNF-YC2*	768	256	28.4	5.11
*ShNF-YC3*	690	230	25.6	4.79
*ShNF-YC4*	831	277	30.9	5.06
*ShNF-YC6*	609	203	21.5	5.37
*ShNF-YC9*	555	185	19.9	5.5

**Fig. 2:**
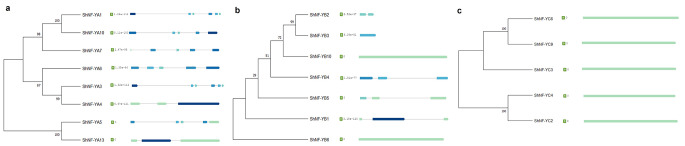
Phylogenetic analysis. Neighbour-joining trees were constructed using the amino acid MSAs of the coding domain regions for **a**) NF-YA, **b**) NF-YB and **c**) NF-YC genes from sugarcane using MEGA 11. Bootstrap values were set to 1000. The predicted intron/exon structure of each gene is visually represented next to the genes, based on the Phytozome data reported from nucleotide BLAST searches against *S. bicolor*. In each case, the best hit e-value is displayed. Blue and green arrows depict exons, and lines depict introns

Multiple protein sequence alignments for each of the identified ShNF-Y family members were used to develop neighbour-joining phylogenetic trees (Fig. 2A-C), also displaying the predicted intron/exon structure of each gene (Fig. 2A- C). NF-YA genes showed between two and five introns, NF-YB showed a maximum of two introns and NF-YC showed no introns in their gene structures.

### Comparative phylogenetic analysis

**Fig. 3:**
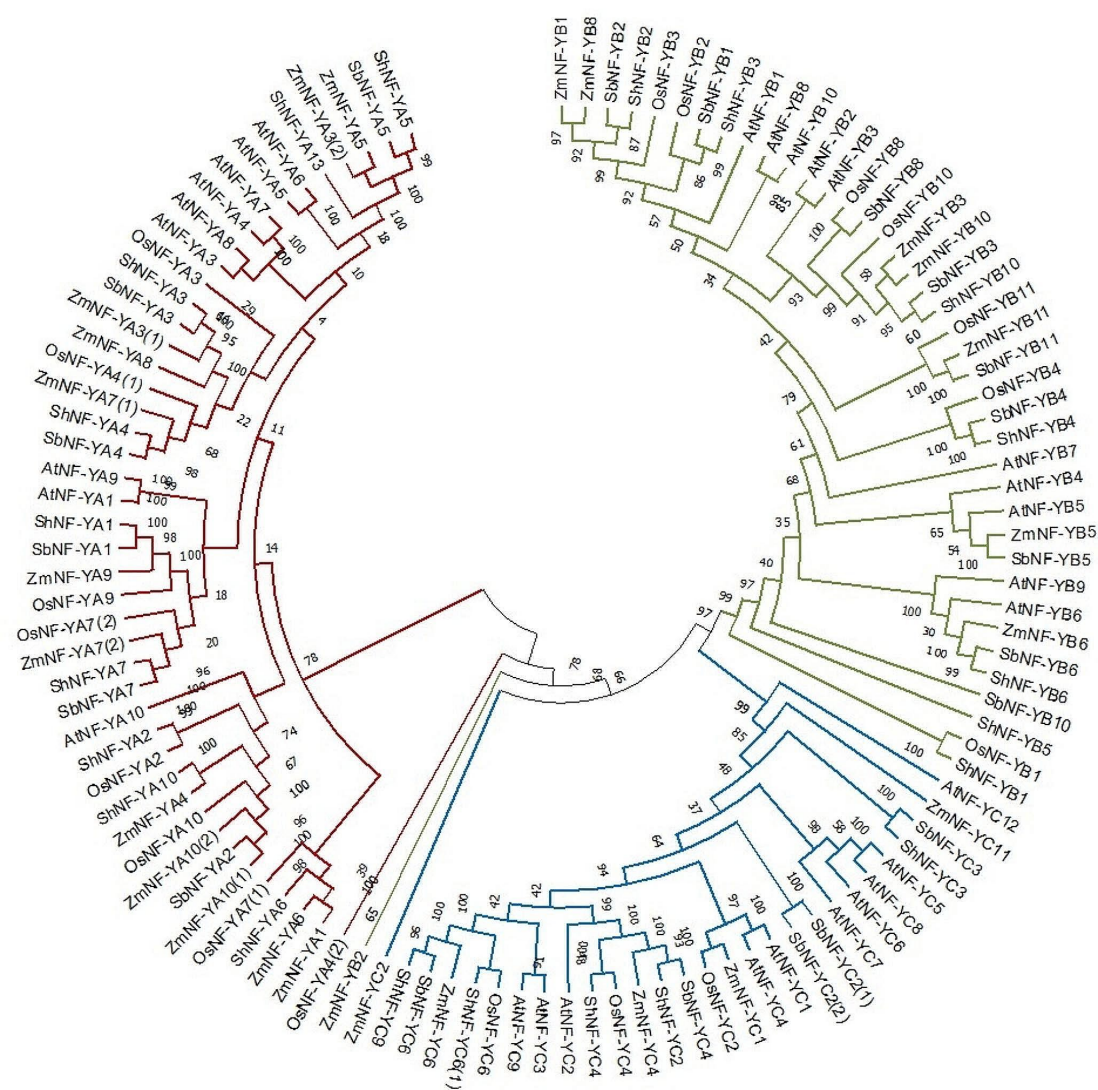
Phylogenetic analysis. Neighbour-joining tree constructed using the amino acid multi sequence alignment of the coding domain regions for all available *NF-Y* genes from sugarcane, sorghum, maize, rice and Arabidopsis using MEGA 11. Bootstrap values were set to 1000. NF-YA proteins indicated in red, NF-YB indicated in green and NF-YC indicated in blue

The potential evolutionary relationships between the various *NF-Y* genes across multiple plant species were assessed based on amino acid similarities (Fig. 3). For this, the amino acid sequences of all available *NF-YA, B* and *C* genes were collected for *A. thaliana, S. bicolor, Z. mays, O. sativa* and *Saccharum* spp. hybrid and included in a multi sequence alignment. The NF-Y proteins clearly grouped into three major clades corresponding to the three subunit family groups. The phylogenetic tree suggests that NF-YB evolved from NF-YA, and that NF-YB and NF-YC are closely related, which is also supported by earlier studies (Malviya et al. 2016). In most cases, the sugarcane proteins show a closer evolutionary relationship to the other monocots (sorghum, maize, and rice) than with the dicot (Arabidopsis) proteins. This is demonstrated by the tendency for the Arabidopsis proteins to form separate smaller clades and the three monocot species to group into clades more interchangeably. This finding is consistent with the study by Chen et al. (2018), who stated that the functions of the NF-Y gene family diverged before the monocots and dicots diverged from one another. Furthermore, a higher number of sugarcane proteins tend to cluster together with sorghum orthologues than to that of rice or maize. This supports previous data that describes an average identity of 91.6% in the genome coding regions between sugarcane and sorghum, compared to an average identity of only 71% in the coding regions between sugarcane and rice (Garsmeur et al. 2018). There is a larger co-linearity between sugarcane and sorghum than either of the two shared with maize or rice, and sorghum has been popular as a close diploid reference for the sugarcane genome, which is not yet nearly fully annotated (Souza et al. 2011; Thirugnanasambandam et al. 2018).

Furthermore, the distribution of the proteins indicates potential discrepancies present in the numeric names of some of the NF-Y putative orthologs across the different species. For example, ZmNF-YA5, shows a high similarity to SbNF-YA5 and ShNF-YA5, but not to OsNF-YA5 or AtNF-YA5. Similarly, the ZmNF-YB8 protein appears distinct from the NF-YB8 protein in other species, which cluster together. Similar discrepancies are observed for NF-YA1 across species. In addition, the ShNF-YB5 protein seems to be distinct from the B5 proteins in the other monocot species, while the ShNF-YB1 protein cluster together with the rice B1 protein but away from the maize, sorghum or Arabidopsis B1 proteins. Notably, all the subunit proteins, NF-YA, B and C group together, the only exception is the maize NF-YB2 and C2 proteins, which fall outside the main subunit clusters. Furthermore, Clarancia et al. (2023) confirmed 16 and 15 orthologous gene pairs between sugarcane and sorghum, and sugarcane and Arabidopsis, respectively.

The ShNF-Y annotations, structural analysis, phylogenies, and ortholog predictions provide a useful starting point for the functional annotation of the ShNF-Y gene family in sugarcane. Past research has indicated that NF-Y genes play roles in a great variety of plant developmental responses, including flowering, root formation and embryogenesis, as well as in abiotic tolerance (drought, cold, heat or flooding (Han et al. 2013; Soyano et al. 2013; Yang et al. 2017; Zhao et al. 2017; Chen et al. 2018). The roles of the ShNF-Y proteins can be predicted to some extent by comparison with functionally annotated NF-Y proteins across species. From the phylogenetic analysis conducted in this study (Fig. 3), ShNF-YA2, ShNF-YA7 and ShNF-YA10 cluster together with OsNF-YA2, OsNF-YA7 and OsNF-YA10, respectively, which are known to regulate drought stress responses in rice (Petroni et al. 2012; Chen et al. 2015; Lee et al. 2015). ShNF-YA4 shows high similarity to OsNF-YA4, which is known to regulate cold stress tolerance in rice (Lee et al. 2015). Furthermore, ShNF-YB3, ShNF-YB4 and ShNF-YB10 clustered together with OsNF-YB2, OsNF-YB4 and SbNF-YB10, respectively, and these genes are known to all contribute to chloroplast biogenesis in rice and sorghum (Petroni et al. 2012; Malviya et al. 2016). ShNF-YB3 is also a likely ortholog of AtNF-YB1, a candidate for functional similarity to this Arabidopsis drought stress regulator (Nelson et al. 2007; Zhao et al. 2017). Additionally, ShNF-YB2 also shows high similarity to SbNF-YB2, which regulates floral meristem development in sorghum (Malviya et al. 2016).

### Expression analysis under abiotic stress

**Fig. 4:**
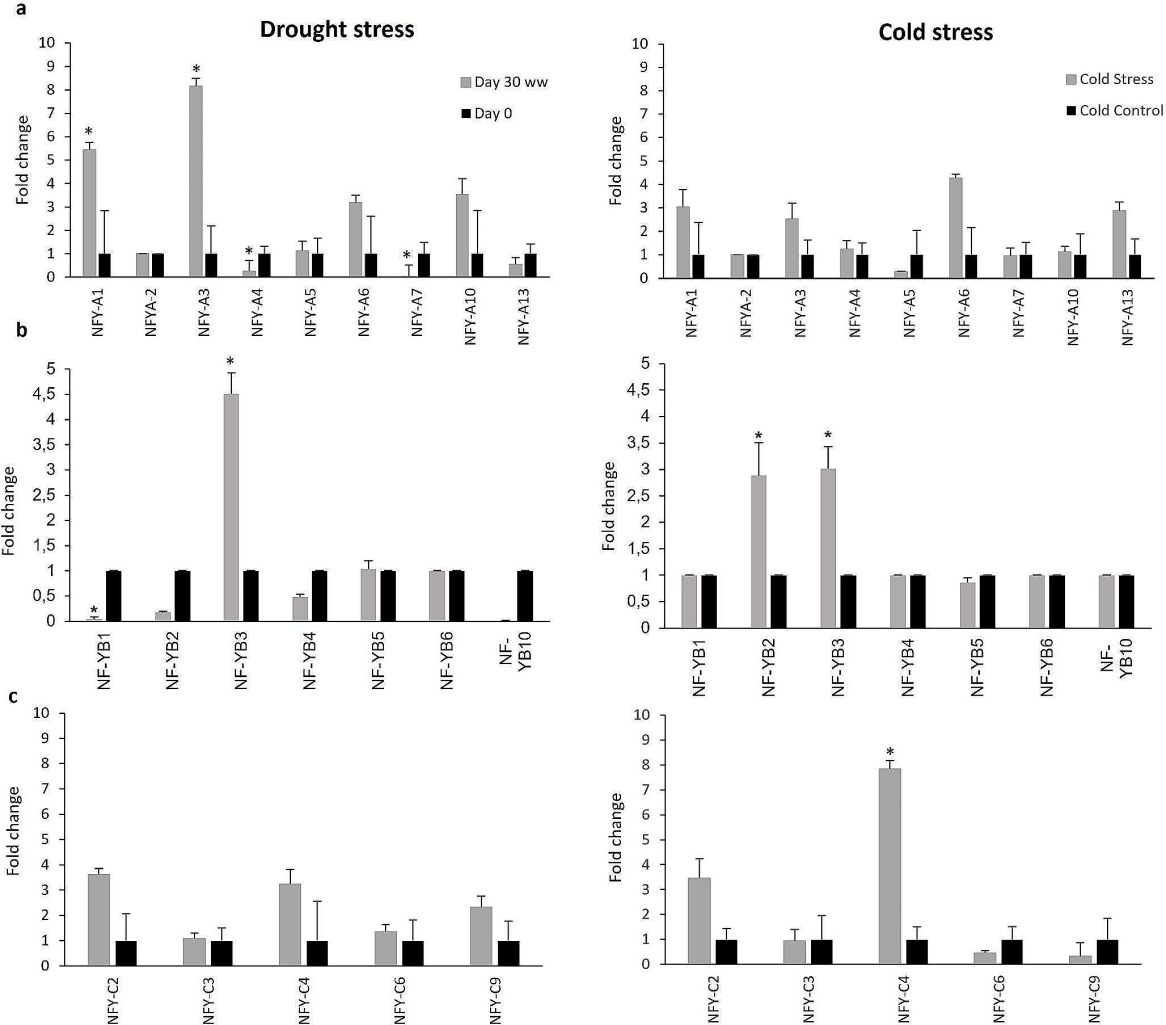
Expression profile of *ShNF-Y* genes, **a**) NF-YA, **b**) NF-YB and **c**) NF-YC, in sugarcane plants subjected to drought and cold stress. The transcript levels of each gene in the stress-treated plants were compared to unstressed control plants, calculated as ∆∆Ct values. The transcript level of the *GAPDH* gene was used as reference. Error bars based on standard error to the mean values obtained from four biological (*n* = 4) and three technical replicates. Asterisks indicated that the expression level was significantly different from the value of the untreated control (* *P* ≤ 0.05)

Gene expression data could offer a further step toward determining the NF-Y genes’ functional roles in this crop. To generate a starting expression dataset, we developed primer sets for each ShNF-Y suitable for quantitative real time PCR (qPCR; Table S1). For each gene, qPCR data was normalized with the *GAPDH* reference gene. When we examined the ShNF-Y gene expression in sugarcane exposed to water deficit stress conditions, three genes *NF-YA1* and *A3* and *B3* were upregulated and two genes, *NF-YA4* and *A7* were down-regulated (Fig. 4). These findings are similar to results reported by Zhang et al. (2016) and Yang et al. (2017), where some NF-Y genes in maize and rice were induced by drought stress (maize: *ZmNF-YB2, 4, 8, 10, 13*, and *16* and *ZmNF-YC6, 8*, and *15*; rice: OsNF-YA4 and B3), while others (maize: *ZmNF-YA1, 3, 4, 6, 7, 10, 12*, and 13, *ZmNF-YB15*, and *ZmNF-YC3* and *9*; rice: *OsNF-YA1, A10* and *OsNF-YB8*) were suppressed. However, in contrast with the microarray expression analysis data reported by Zhang et al. (2016), Cao et al. (2023) reported that the *ZmNF-YA12* gene played an important role in drought tolerance. When this gene was silenced in maize, it resulted in reduced photosynthesis, decreased chlorophyll content and antioxidant scavenging activity. This contrasting result underlines the importance of confirming gene function through *in planta* experimentation. On the other hand, like the initial gene expression analysis done by Zhang et al. (2016) mentioned above, Wang et al. (2018) confirmed the role of the *ZmNF-YB16* gene in plants’ drought stress response when they overexpressed this gene in maize, and the transgenic plants displayed improved drought stress tolerance by maintaining higher photosynthesis in the transgenic plants. Furthermore, nine NF-Y genes in wheat also appeared to be responsive to drought stress (Stephenson et al. 2007). Noteworthy, in the current study the *ShNF-YA7* gene was downregulated under water deficit stress, which contrasts with results reported by Lee et al. (2015), linking the predicted rice *NF-YA7* ortholog with enhanced drought tolerance. In a recent study conducted by Clarancia et al. (2023), two sugarcane *NF-YA* genes (NF-YA3 and A5; CDS of 516 and 915 bp respectively) were also upregulated under drought stress.

It has also been suggested that NF-Y TFs is involved in the cold stress response in plants (Zhang et al. 2023). When sugarcane was exposed to cold stress, three genes were significantly upregulated, namely *ShNF-YB2, B3* and *C4* (Fig. 4). This is in contrast with two rice genes, *OsNF-YA1* and *A5*, that were downregulated when the rice plants were exposed to cold (Yang et al. 2017). In sorghum the *NF-YA8* gene was linked to cold stress (Maheshwari et al. 2019).

### Expression analysis under biotic stress

The role of NF-Y genes in biotic stress tolerance has been documented to some extent. For example, it is known that the *ZmNF-YA3, ZmNF-YA8* and *ZmNF-YA12* (Zhang et al. 2016), and *ZmNF-YA1* and *ZmNF-YA6* (Lv et al. 2022) genes are upregulated after infection by fungal pathogens. Furthermore, through miR169-mediated regulation, studies conducted in rice and Arabidopsis have indicated that NF-YA genes, specifically *NF-YA3* and *NF-YA10*, play a positive role in pathogen disease resistance (Li et al. 2017). Also, through gene manipulation, Di Martino (2018) revealed the crucial role of *NF-YA2* and overlapping functionality between *NF-YB2* and *NF-YB3* genes in the regulation of defense responses in rice against *Botrytis cinerea*.

The ShNFY gene sequences identified in this study were appended to a transcriptome constructed to investigate the effects of eldana infestation in sugarcane. Eldana is a Lepidoptera insect pest of sugarcane, widely distributed across Africa. In South Africa, eldana was recorded as early as 1939 and again in 1970 in cultivars such as POJ2725 and NCo376 as a pest of sugarcane (Carnegie 1982), and since these early recordings, it now occurs across most sugarcane cultivation areas in the country. Classed as stem borers, these insects cause major yield losses, reported to be as much as US$ 90 million in South Africa (Rutherford 2015). These insects damage stalks resulting in lower sucrose, higher fibre and lower stalk mass and stalk length (Goebel and Way 2003).

**Fig. 5:**
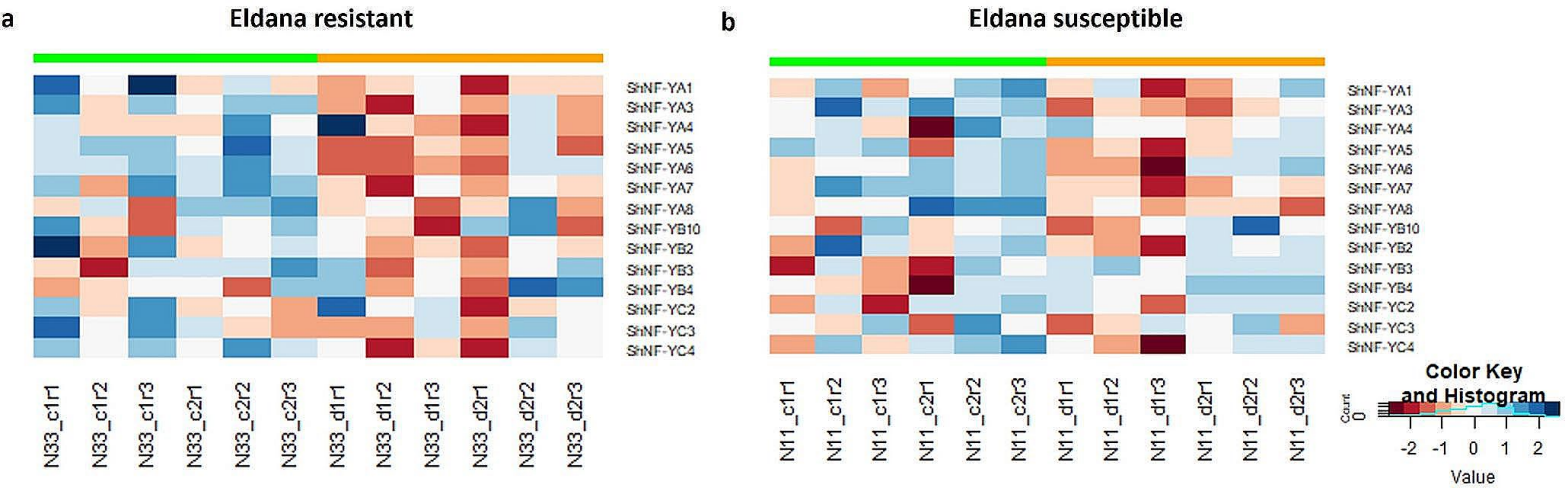
Expression profile of ShNF-Y transcripts in the **a**) N33 (eldana resistant) and **b**) N11 (eldana susceptible) *Saccharum* hybrid cultivars. Eldana-challenged (d) and unchallenged controls (c) were sampled at two time points (d1: ∼24 h after the challenge, or d2: ∼72 h after the challenge) compared to their unchallenged controls (c1: 1 day after challenge; c2: 3 days after challenge). The relative gene-level variation is visualized by plotting z-scores of the log transformed TPM. The largest gene expression values are displayed in blue, intermediate values in white and the smallest values in red

An RNA-seq dataset consisting of 24 samples was constructed to compare the response in two *Saccharum* hybrid cultivars (N33: eldana resistant; N11: eldana susceptible) to an eldana challenge compared to their unchallenged controls at two timepoints (1 and 3 days after challenge). The transcript level abundance (transcript per million; TPM) estimates were summed for a gene-level analysis. A heatmap was created plotting the relative gene-level variation in expression of the *NF-Y* genes, which highlights the relative abundance of *NF-Y* genes in each sample pool of transcripts (Fig. 5; Supplementary dataset S3). Read support was obtained for seven *NF-YA*, four *NF-YB* and three *NF-YC* genes, respectively. In general, relative abundance levels of *Saccharum* hybrid *NF-YA* genes were lower in the eldana-challenged samples than in the unchallenged control samples (Fig. 5A and B).

Furthermore, outliers replicate samples (N33_d2r1, N11_c1r1 and N11_d1r3) were identified using principal component analysis. These samples were removed as the variance could be indicative of technical variability rather than biological variability. This was done through sleuth analysis, which is a lightweight tool that uses transcript abundance estimates from pseudo-alignment algorithms based on bootstrap sampling to perform differential expression analysis of transcripts. The sleuth results were interrogated to extract the *ShNF-Y* genes and transcripts (Supplementary data tables S4-7) and the differentially expressed *ShNF-Y* genes (q value < 0.05) were extracted in Table 3. At the gene level, *ShNF-YA6, ShNF-YA3* and *ShNF-YA7* were downregulated in the early resistant (N33) response to eldana challenge, whilst only *ShNF-YA6* was downregulated in the susceptible (N11) early response. The most downregulated transcript was one encoding *ShNF-YA3* in the early resistant response (downregulated 3.59 log2FC). No differential expression of *ShNFY* genes were identified in either the resistant (N33) or the susceptible (N11) late response to the challenge. This is a first report potentially linking NF-YA genes to the biotic stress response in sugarcane associated with insect tolerance.

**Table 3:** *ShNF-Y* genes identified as differentially expressed in the early response between eldana challenged samples and the unchallenged controls. Cultivars N33 and N11 considered resistant and susceptible, respectively

Condition	Gene	qval	Most significant transcript	Log_2_FC of most significant transcript
*N11 Early Response*				
	ShNF-YA6	0.02365	ShNF-YA6	-1.18
*N33 Early Response*				
	ShNF-YA6	1.57E-08	ShNF-YA6	-1.38
	ShNF-YA3	0.000912	TRINITY_DN226448_c0_g1_i1	-3.59
	ShNF-YA7	0.038219	TRINITY_DN230864_c0_g5_i2	-0.90

To conclude, the ShNF-Y family of TFs represents an understudied group of proteins in sugarcane with predicted roles ranging from growth and development to defence response metabolism in the plant. Ongoing research highlight the potential of NF-Y genes in mitigating plant stress responses across plant species. This study identified and characterized 21 ShNF-Y genes in sugarcane, elucidating their gene structures and phylogenetic relationships with NF-Y proteins from other Poaceae species. Nomenclature for these identified genes was proposed based on database accessions and ortholog identification in other grass species. Additionally, this study reports on the differential expression patterns of these TFs, shedding light on their involvement in drought, cold, and biotic stress responses. *In silico* and qPCR-based gene expression analyses have provided valuable insights into the roles of these proteins in various environmentally and biotic stress responses. Overall, this data offers meaningful insights into ShNF-Ys and lays the groundwork for further functional studies of NF-Y genes in sugarcane, which could ultimately contribute to the development of commercial plant varieties with enhanced stress tolerance, leveraging these TFs as molecular switches to manipulate downstream stress responses.

### Electronic supplementary material

Below is the link to the electronic supplementary material.

Supplementary Material 1
