# Supplementary material for: Identification and expression analysis of nuclear factor Y transcription factor genes under drought, cold and Eldana infestation in sugarcane (Saccharum spp. hybrid)
Source: Genes Genomics. 2024 Jun 14;46(8):927–40. doi: 10.1007/s13258-024-01529-3 (PMC11329523; doi:10.1007/s13258-024-01529-3)
Supplement: Supplementary file 1 — Supplementary Material 1 [file 13258_2024_1529_MOESM1_ESM.docx]

**Supplementary Data**

**Table S1.** Primer sequences used for qPCR analysis of the NF-Y genes.

| **Primer name** | **5' - 3'** | **Gene** | **Amplicon size** |
| --- | --- | --- | --- |
| QUBQ1 F | AGCCTCAGACCAGATTCCAA | *Ubiquitin* | 110 |
| QUBQ1 R | AATCGCTGTCGAACTACTTGC |  |  |
| QGAPDH F | TTGGTTTCCACTGACTTCGTT | *GAPDH* | 122 |
| QGAPDH R | CTGTAGCCCCACTCGTTGT |  |  |
| QACTIN F | CTCAACCCCAAGGCTAACAG | *Actin* | 195 |
| QACTIN R | GGCATGAGGAAGGGCATAA |  |  |
| NFYA1 Fwd | GGTGCAGCCGTGGTG | *ShNFYA1* | 104 |
| NFYA1 REV | CCGGGCTGCTCGACA |  |  |
| NFYA2 Fwd | CCGGGTCTCCGGGAA | *ShNFYA2* | 111 |
| NFYA2 Rev | CCGTCGCGGCTCCG |  |  |
| NFYA3 Fwd | GGCTCCTTGTCAGGAAATGG | *ShNFYA3* | 104 |
| NFYA3 Rev | GCTGATGCTTCTTGGTGAGG |  |  |
| NFYA4 Fwd | CATCCCATGCCCAACTATGA | *ShNFYA4* | 113 |
| NFYA4 Rev | TGCCGAGACTGCTCAGAT |  |  |
| NFYA5 Fwd | AGAGGAAGGGACGGCA | *ShNFYA5* | 101 |
| NFYA5 Rev | CGAAGAGGGGAGAAGCATG |  |  |
| NFYA6 Fwd | GGTCATCAAATGCCTGATTACGA | *ShNFYA6* | 100 |
| NFYA6 Rev | TATGCTCGTTGAGGCTTCCT |  |  |
| NFYA7 Fwd | GTGTTTCAGGTGACCACAGG | *ShNFYA7* | 100 |
| NFYA7 Rev | TGGCTATCTGAACTAGTAAC |  |  |
| NFYA8 Fwd | GTGAAGACATGGATCACTACCAC | *ShNFYA8* | 101 |
| NFYA8 Rev | GTTACCAACGACCCCATGC |  |  |
| NFYA13 Fwd | CTCTACAGGCACCAGCG | *ShNFYA13* | 101 |
| NFYA13 Rev | ACAAGGTGTATATATGATGGACGG |  |  |
| NF-YB1 F | CCGCAAGGCGAAGCAAT | *NF-YB1* | 125 |
| NF-YB1 R | CGGGCTGCCGATGG |  |  |
| NF-YB2 F  NF-YB2 R | CCCCTCAAGGTGTACCTG  CCTTGGTCATATGGGAG | *NF-YB2* | 115 |
| NF-YB3 F  NF-YB3 R | AGGATGCAATCAGTTCCCAT  TTGTGGTACTGTGGCTGCAT | *NF-YB3* | 106 |
| NF-YB4 F  NF-YB4 R | GGGTGACACAAAGGGTTCAA  TGAATTTGTACAGGGTTTACTTCGC | *NF-YB4* | 148 |
| NF-YB5 F  NF-YB5 R | AGTGGTGGCACTCAACAACA  TTCAGGGGAAACGAGCAGTAA | *NF-YB5* | 106 |
| NF-YB6 F  NF-YB6 R | CGCCATGGCCGCGTA  GGCTTCGAGCACCCGCA | *NF-YB6* | 116 |
| NF-YB10 F  NF-YB10 R | AACGCCAAGATCTCCAAGGA  GGAACTTGTGGAGGTAGAGCT | *NF-YB10* | 102 |
| NFYC2 Fwd | TGCAATGGGTGTTGCTGC | *ShNFYC2* | 103 |
| NFYC2 Rev | CTGTAAACCAGCAGATGCAACT |  |  |
| NFYC3 Fwd | TCCCTATGCTGACTACTACAG | *ShNFYC3* | 100 |
| NFYC3 Rev | TCTTGGGTCATCACAGGTCA |  |  |
| NFYC4 Fwd | TTACTATGTACCAGCACAGCAG | *ShNFYC4* | 101 |
| NFYC4 Rev | GCCCTTGCGGCTTCTG |  |  |
| NFYC6 Fwd | GCACCGAGGTGTTCGAC | *ShNFYC6* | 115 |
| NFYC6 Rev | GCAGGACGCGGGATC |  |  |
| NFYC9 Fwd | GCGGGACGAGGCCAA | *ShNFYC9* | 106 |
| NFYC9 Rev | ACTGTGGCTGGACGTAGT |  |  |


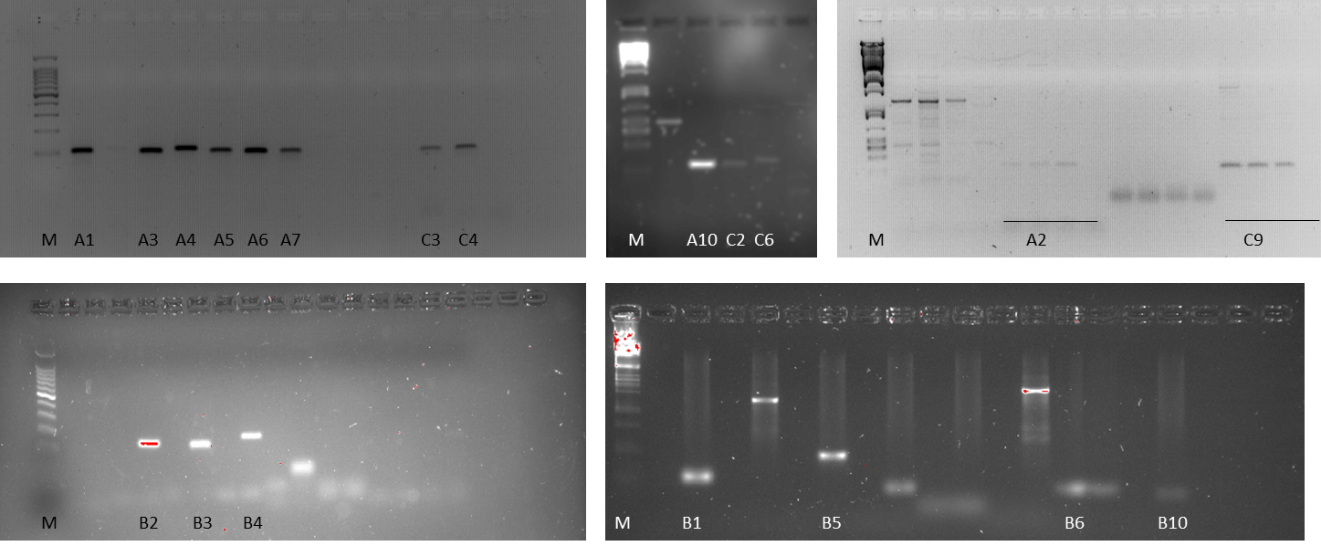


**Figure S2**: Conventional PCR analysis to confirm the amplification of single amplicons for each NF-Y gene (NF-Y A1 to A13; B1 to B10; and C2 tot C9). In some instances, multiple primer pairs had to be designed for a specific gene to eventually result in successful amplification (listed in Table S1). Open lanes on the gel images indicate unsuccessful amplification.

**Table S3:** Transcript table of the transcripts making up the ShNFY genes showing in depth summary statistics and test results for each individual transcript.

| target_id | gene | pval | qval | b (log2 fold change) |
| --- | --- | --- | --- | --- |
| TRINITY_DN226448_c0_g1_i1 | ShNF-YA3 | 0.00024 | 0.01343 | -2.82186 |
| ShNF-YA6 | ShNF-YA6 | 0.00101 | 0.03627 | -0.93525 |
| TRINITY_DN218789_c0_g1_i1 | ShNF-YA6 | 0.00858 | 0.13617 | -0.73651 |
| TRINITY_DN226448_c0_g1_i6 | ShNF-YA3 | 0.0097 | 0.14532 | -2.08742 |
| TRINITY_DN222879_c1_g5_i6 | ShNF-YA1 | 0.0187 | 0.20316 | -0.5973 |
| ShNF-YA7 | ShNF-YA7 | 0.02361 | 0.22773 | -0.50546 |
| TRINITY_DN230864_c0_g5_i2 | ShNF-YA7 | 0.03808 | 0.29014 | -0.64345 |
| TRINITY_DN218789_c0_g1_i5 | ShNF-YA6 | 0.06268 | 0.36521 | -0.79456 |
| TRINITY_DN238653_c0_g4_i2 | ShNF-YC2 | 0.10048 | 0.45147 | 0.329432 |
| TRINITY_DN232303_c1_g1_i4 | ShNF-YA4 | 0.11698 | 0.48144 | 0.638742 |
| TRINITY_DN236822_c1_g1_i1 | ShNF-YB4 | 0.12728 | 0.4973 | 0.577857 |
| TRINITY_DN222879_c1_g5_i2 | ShNF-YA1 | 0.14206 | 0.52045 | -0.31485 |
| TRINITY_DN218789_c0_g1_i6 | ShNF-YA6 | 0.14887 | 0.53007 | -0.9778 |
| TRINITY_DN220412_c0_g2_i2 | ShNF-YC4 | 0.14907 | 0.53047 | -1.02395 |
| TRINITY_DN233450_c0_g2_i1 | ShNF-YA8 | 0.15773 | 0.54175 | -0.52879 |
| ShNF-YB2 | ShNF-YB2 | 0.16892 | 0.55726 | -0.29087 |
| TRINITY_DN226448_c0_g1_i5 | ShNF-YA3 | 0.17773 | 0.56791 | -0.93173 |
| TRINITY_DN220412_c0_g2_i1 | ShNF-YC4 | 0.21095 | 0.60512 | -0.27685 |
| TRINITY_DN232303_c1_g1_i1 | ShNF-YA4 | 0.23778 | 0.63382 | -0.96547 |
| ShNF-YC4 | ShNF-YC4 | 0.29594 | 0.68358 | -0.21456 |
| ShNF-YA3 | ShNF-YA3 | 0.30429 | 0.69021 | -0.41664 |
| TRINITY_DN218789_c0_g1_i17 | ShNF-YA6 | 0.31461 | 0.69899 | -0.84256 |
| ShNF-YC2 | ShNF-YC2 | 0.3253 | 0.70787 | -0.90349 |
| TRINITY_DN226448_c0_g1_i11 | ShNF-YA3 | 0.39031 | 0.75526 | -0.56318 |
| TRINITY_DN222879_c1_g5_i4 | ShNF-YA1 | 0.40921 | 0.76659 | -0.29988 |
| TRINITY_DN218159_c0_g1_i3 | ShNF-YB3 | 0.54578 | 0.8444 | 0.16356 |
| TRINITY_DN222879_c1_g5_i3 | ShNF-YA1 | 0.61657 | 0.87697 | -0.17194 |
| ShNF-YB3 | ShNF-YB3 | 0.64515 | 0.88878 | 0.133869 |
| TRINITY_DN236822_c1_g1_i3 | ShNF-YB4 | 0.74141 | 0.9252 | 0.173365 |
| ShNF-YA4 | ShNF-YA4 | 0.81546 | 0.95032 | 0.047876 |
| TRINITY_DN238653_c0_g4_i1 | ShNF-YC2 | 0.86837 | 0.96547 | -0.08345 |
| TRINITY_DN218789_c0_g1_i13 | ShNF-YA6 | 0.93873 | 0.98479 | 0.092731 |
| TRINITY_DN233450_c0_g2_i3 | ShNF-YA8 | 0.96144 | 0.9903 | -0.0184 |

**Table S4:** Sleuth results for the N33 early response

| **target_id** | **gene_id** | **pval** | **qval** | **b (log2FC)** | **se_b** |
| --- | --- | --- | --- | --- | --- |
| ShNF-YA6 | ShNF-YA6 | 1.86E-08 | 8.20E-06 | -1.37649 | 0.244723 |
| TRINITY_DN226448_c0_g1_i1 | ShNF-YA3 | 4.81E-05 | 0.004281 | -3.59045 | 0.883364 |
| TRINITY_DN218789_c0_g1_i1 | ShNF-YA6 | 0.000126 | 0.008536 | -1.08786 | 0.283667 |
| TRINITY_DN226448_c0_g1_i6 | ShNF-YA3 | 0.001984 | 0.051409 | -2.85739 | 0.92395 |
| TRINITY_DN230864_c0_g5_i2 | ShNF-YA7 | 0.010726 | 0.13256 | -0.90492 | 0.354663 |
| TRINITY_DN238653_c0_g4_i2 | ShNF-YC2 | 0.013112 | 0.148018 | 0.525172 | 0.211702 |
| ShNF-YA7 | ShNF-YA7 | 0.031844 | 0.234784 | -0.54553 | 0.254166 |
| TRINITY_DN218789_c0_g1_i6 | ShNF-YA6 | 0.060797 | 0.321779 | -1.45532 | 0.776185 |
| TRINITY_DN218789_c0_g1_i5 | ShNF-YA6 | 0.066961 | 0.336949 | -0.90561 | 0.494344 |
| TRINITY_DN220412_c0_g2_i1 | ShNF-YC4 | 0.084912 | 0.378247 | -0.43473 | 0.252329 |
| TRINITY_DN218789_c0_g1_i17 | ShNF-YA6 | 0.089063 | 0.386002 | -1.56004 | 0.917474 |
| TRINITY_DN232303_c1_g1_i4 | ShNF-YA4 | 0.092169 | 0.392454 | 0.793543 | 0.471206 |
| TRINITY_DN233450_c0_g2_i1 | ShNF-YA8 | 0.09267 | 0.393242 | -0.72764 | 0.432739 |
| TRINITY_DN226448_c0_g1_i5 | ShNF-YA3 | 0.103539 | 0.413354 | -1.32369 | 0.813108 |
| TRINITY_DN222879_c1_g5_i6 | ShNF-YA1 | 0.128487 | 0.455507 | -0.45463 | 0.299079 |
| TRINITY_DN232303_c1_g1_i1 | ShNF-YA4 | 0.1814 | 0.53081 | -1.31532 | 0.984186 |
| TRINITY_DN222879_c1_g5_i2 | ShNF-YA1 | 0.209887 | 0.566282 | -0.3327 | 0.265337 |
| ShNF-YC2 | ShNF-YC2 | 0.292681 | 0.649007 | -1.1804 | 1.12178 |
| ShNF-YA3 | ShNF-YA3 | 0.296054 | 0.65271 | -0.48518 | 0.464322 |
| TRINITY_DN236822_c1_g1_i1 | ShNF-YB4 | 0.309822 | 0.664466 | 0.439255 | 0.43251 |
| TRINITY_DN226448_c0_g1_i11 | ShNF-YA3 | 0.355077 | 0.70306 | -0.69333 | 0.74972 |
| ShNF-YC4 | ShNF-YC4 | 0.360473 | 0.706556 | -0.21367 | 0.23366 |
| ShNF-YB2 | ShNF-YB2 | 0.369533 | 0.7139 | -0.23222 | 0.258787 |
| TRINITY_DN218789_c0_g1_i13 | ShNF-YA6 | 0.404994 | 0.739693 | -1.05745 | 1.269847 |
| TRINITY_DN218159_c0_g1_i3 | ShNF-YB3 | 0.407462 | 0.7414 | 0.257225 | 0.310521 |
| TRINITY_DN220412_c0_g2_i2 | ShNF-YC4 | 0.434038 | 0.758098 | -0.64173 | 0.820308 |
| TRINITY_DN222879_c1_g5_i4 | ShNF-YA1 | 0.463576 | 0.775818 | -0.30586 | 0.41729 |
| ShNF-YA4 | ShNF-YA4 | 0.560555 | 0.830434 | 0.142943 | 0.245599 |
| TRINITY_DN238653_c0_g4_i1 | ShNF-YC2 | 0.613178 | 0.857485 | -0.29008 | 0.573791 |
| TRINITY_DN222879_c1_g5_i3 | ShNF-YA1 | 0.667253 | 0.88203 | 0.169979 | 0.395372 |
| ShNF-YB3 | ShNF-YB3 | 0.824306 | 0.94507 | 0.074128 | 0.333895 |
| TRINITY_DN236822_c1_g1_i3 | ShNF-YB4 | 0.870633 | 0.959812 | -0.09754 | 0.598924 |
| TRINITY_DN233450_c0_g2_i3 | ShNF-YA8 | 0.913389 | 0.973785 | -0.0512 | 0.470756 |

**Table S5:** Sleuth results for the N33 late response

| **target_id** | **gene_id** | **pval** | **qval** | **b (log2FC)** | **se_b** |
| --- | --- | --- | --- | --- | --- |
| TRINITY_DN222879_c1_g5_i6 | ShNF-YA1 | 0.018812 | 0.34613 | -0.8113 | 0.345347 |
| TRINITY_DN220412_c0_g2_i2 | ShNF-YC4 | 0.091738 | 0.666947 | -1.59728 | 0.94721 |
| TRINITY_DN226448_c0_g1_i1 | ShNF-YA3 | 0.101793 | 0.691004 | -1.66898 | 1.020021 |
| TRINITY_DN236822_c1_g1_i1 | ShNF-YB4 | 0.115638 | 0.720122 | 0.78576 | 0.499419 |
| ShNF-YA7 | ShNF-YA7 | 0.129145 | 0.747652 | -0.44536 | 0.293486 |
| TRINITY_DN222879_c1_g5_i3 | ShNF-YA1 | 0.133612 | 0.755416 | -0.68481 | 0.456536 |
| ShNF-YB2 | ShNF-YB2 | 0.204879 | 0.851076 | -0.37884 | 0.298822 |
| TRINITY_DN218789_c0_g1_i13 | ShNF-YA6 | 0.215028 | 0.861042 | 1.817997 | 1.466293 |
| TRINITY_DN218789_c0_g1_i5 | ShNF-YA6 | 0.271275 | 0.906852 | -0.62798 | 0.57082 |
| ShNF-YA6 | ShNF-YA6 | 0.333311 | 0.93656 | -0.27339 | 0.282582 |
| TRINITY_DN222879_c1_g5_i2 | ShNF-YA1 | 0.347094 | 0.942585 | -0.28808 | 0.306385 |
| TRINITY_DN226448_c0_g1_i6 | ShNF-YA3 | 0.382111 | 0.956873 | -0.93247 | 1.066886 |
| TRINITY_DN236822_c1_g1_i3 | ShNF-YB4 | 0.401888 | 0.963351 | 0.579718 | 0.691578 |
| ShNF-YC4 | ShNF-YC4 | 0.423637 | 0.969206 | -0.21588 | 0.269807 |
| TRINITY_DN232303_c1_g1_i4 | ShNF-YA4 | 0.454958 | 0.974413 | 0.406539 | 0.544102 |
| TRINITY_DN218789_c0_g1_i1 | ShNF-YA6 | 0.52247 | 0.985883 | -0.20948 | 0.327551 |
| TRINITY_DN230864_c0_g5_i2 | ShNF-YA7 | 0.539547 | 0.989728 | -0.25125 | 0.40953 |
| TRINITY_DN222879_c1_g5_i4 | ShNF-YA1 | 0.546022 | 0.99128 | -0.29091 | 0.481845 |
| ShNF-YA3 | ShNF-YA3 | 0.55834 | 0.992499 | -0.31381 | 0.536153 |
| ShNF-YB3 | ShNF-YB3 | 0.562157 | 0.99258 | 0.22348 | 0.385549 |
| TRINITY_DN233450_c0_g2_i1 | ShNF-YA8 | 0.644557 | 0.997146 | -0.23052 | 0.499684 |
| TRINITY_DN226448_c0_g1_i11 | ShNF-YA3 | 0.670813 | 0.998439 | -0.36795 | 0.865702 |
| TRINITY_DN226448_c0_g1_i5 | ShNF-YA3 | 0.714241 | 0.999232 | -0.34379 | 0.938896 |
| ShNF-YA4 | ShNF-YA4 | 0.738367 | 0.999232 | -0.09472 | 0.283593 |
| TRINITY_DN232303_c1_g1_i1 | ShNF-YA4 | 0.698181 | 0.999232 | -0.44069 | 1.13644 |
| TRINITY_DN218789_c0_g1_i17 | ShNF-YA6 | 0.825448 | 0.999232 | 0.233645 | 1.059408 |
| TRINITY_DN218789_c0_g1_i6 | ShNF-YA6 | 0.770445 | 0.999232 | -0.26152 | 0.896262 |
| TRINITY_DN218159_c0_g1_i3 | ShNF-YB3 | 0.948714 | 0.999232 | 0.023063 | 0.358558 |
| ShNF-YC2 | ShNF-YC2 | 0.706292 | 0.999232 | -0.48813 | 1.29532 |
| TRINITY_DN238653_c0_g4_i1 | ShNF-YC2 | 0.732475 | 0.999232 | 0.226485 | 0.662556 |
| TRINITY_DN238653_c0_g4_i2 | ShNF-YC2 | 0.883491 | 0.999232 | 0.035823 | 0.244452 |
| TRINITY_DN220412_c0_g2_i1 | ShNF-YC4 | 0.890709 | 0.999232 | -0.04004 | 0.291364 |
| TRINITY_DN233450_c0_g2_i3 | ShNF-YA8 | 0.954793 | 0.999331 | 0.030815 | 0.543582 |

**Table S6:** Sleuth results for the N11 early response

| **target_id** | **gene_id** | **pval** | **qval** | **b (log2FC)** | **se_b** |
| --- | --- | --- | --- | --- | --- |
| ShNF-YA6 | ShNF-YA6 | 0.000569 | 0.044667 | -1.18227 | 0.343072 |
| TRINITY_DN218789_c0_g1_i1 | ShNF-YA6 | 0.001656 | 0.09034 | -1.14118 | 0.362759 |
| TRINITY_DN226448_c0_g1_i11 | ShNF-YA3 | 0.04058 | 0.473275 | -2.0988 | 1.024907 |
| ShNF-YA3 | ShNF-YA3 | 0.050929 | 0.517124 | -1.5604 | 0.799353 |
| ShNF-YB2 | ShNF-YB2 | 0.060532 | 0.548736 | -0.68384 | 0.364347 |
| ShNF-YC4 | ShNF-YC4 | 0.067408 | 0.569724 | -0.7514 | 0.410838 |
| TRINITY_DN236822_c1_g1_i3 | ShNF-YB4 | 0.094045 | 0.631887 | 1.687722 | 1.007933 |
| TRINITY_DN222879_c1_g5_i2 | ShNF-YA1 | 0.100846 | 0.644405 | -0.47604 | 0.290133 |
| ShNF-YA7 | ShNF-YA7 | 0.14847 | 0.704099 | -0.64838 | 0.448719 |
| TRINITY_DN236822_c1_g1_i1 | ShNF-YB4 | 0.171935 | 0.725245 | 1.519159 | 1.112111 |
| ShNF-YC2 | ShNF-YC2 | 0.194085 | 0.742851 | -0.99866 | 0.769032 |
| TRINITY_DN218789_c0_g1_i15 | ShNF-YA6 | 0.199288 | 0.746689 | -1.92194 | 1.497328 |
| TRINITY_DN226448_c0_g1_i5 | ShNF-YA3 | 0.20921 | 0.755232 | -1.48775 | 1.184759 |
| TRINITY_DN218789_c0_g1_i9 | ShNF-YA6 | 0.245919 | 0.778639 | -1.5968 | 1.376177 |
| TRINITY_DN218159_c0_g1_i3 | ShNF-YB3 | 0.264241 | 0.78688 | 1.570591 | 1.406807 |
| TRINITY_DN218789_c0_g1_i5 | ShNF-YA6 | 0.270681 | 0.789804 | -1.67078 | 1.516825 |
| TRINITY_DN218789_c0_g1_i13 | ShNF-YA6 | 0.298846 | 0.80424 | -1.8556 | 1.786097 |
| TRINITY_DN238653_c0_g4_i2 | ShNF-YC2 | 0.33832 | 0.821515 | 0.291162 | 0.304089 |
| TRINITY_DN218789_c0_g1_i8 | ShNF-YA6 | 0.366462 | 0.83295 | -1.4616 | 1.618393 |
| TRINITY_DN226448_c0_g1_i6 | ShNF-YA3 | 0.368761 | 0.833831 | 1.310787 | 1.45838 |
| TRINITY_DN218789_c0_g1_i10 | ShNF-YA6 | 0.439405 | 0.858128 | -0.81663 | 1.05617 |
| ShNF-YA5 | ShNF-YA5 | 0.484536 | 0.872621 | -0.95309 | 1.363461 |
| TRINITY_DN222879_c1_g5_i6 | ShNF-YA1 | 0.485062 | 0.872867 | 0.965179 | 1.382414 |
| TRINITY_DN218789_c0_g1_i14 | ShNF-YA6 | 0.517934 | 0.882447 | -1.33281 | 2.061464 |
| TRINITY_DN232303_c1_g1_i1 | ShNF-YA4 | 0.530548 | 0.88687 | -0.56677 | 0.903697 |
| TRINITY_DN233450_c0_g2_i3 | ShNF-YA8 | 0.542648 | 0.891062 | -0.66784 | 1.09696 |
| ShNF-YA4 | ShNF-YA4 | 0.551399 | 0.894029 | 0.260768 | 0.437775 |
| TRINITY_DN220412_c0_g2_i1 | ShNF-YC4 | 0.565326 | 0.898626 | 0.905843 | 1.575521 |
| TRINITY_DN220412_c0_g2_i2 | ShNF-YC4 | 0.578729 | 0.902234 | 0.857701 | 1.544733 |
| ShNF-YB3 | ShNF-YB3 | 0.59996 | 0.908497 | -0.40524 | 0.772689 |
| TRINITY_DN233450_c0_g2_i1 | ShNF-YA8 | 0.644436 | 0.922935 | 0.438728 | 0.950645 |
| TRINITY_DN218789_c0_g1_i17 | ShNF-YA6 | 0.65719 | 0.926944 | -0.74209 | 1.672145 |
| TRINITY_DN218789_c0_g1_i6 | ShNF-YA6 | 0.665263 | 0.928826 | -0.6422 | 1.484323 |
| TRINITY_DN222879_c1_g5_i4 | ShNF-YA1 | 0.73727 | 0.946588 | 0.441057 | 1.31474 |
| TRINITY_DN230864_c0_g5_i2 | ShNF-YA7 | 0.808534 | 0.961569 | -0.17739 | 0.732055 |
| TRINITY_DN222855_c0_g1_i5 | ShNF-YA5 | 0.843471 | 0.96904 | -0.33598 | 1.701548 |
| TRINITY_DN222879_c1_g5_i3 | ShNF-YA1 | 0.948029 | 0.98982 | -0.04958 | 0.76063 |
| TRINITY_DN238653_c0_g4_i1 | ShNF-YC2 | 0.953786 | 0.991163 | -0.06061 | 1.045781 |

**Table S7:** Sleuth results for the N11 late response

| **target_id** | **gene_id** | **pval** | **qval** | **b (log2FC)** | **se_b** |
| --- | --- | --- | --- | --- | --- |
| TRINITY_DN218789_c0_g1_i1 | ShNF-YA6 | 0.025881 | 0.430239 | 0.705477 | 0.316642 |
| TRINITY_DN226448_c0_g1_i5 | ShNF-YA3 | 0.033572 | 0.479603 | -2.19773 | 1.034143 |
| TRINITY_DN236822_c1_g1_i1 | ShNF-YB4 | 0.036523 | 0.496257 | 2.029851 | 0.97073 |
| TRINITY_DN236822_c1_g1_i3 | ShNF-YB4 | 0.052531 | 0.568717 | 1.705714 | 0.879796 |
| ShNF-YA7 | ShNF-YA7 | 0.126222 | 0.731671 | -0.59894 | 0.391674 |
| TRINITY_DN233450_c0_g2_i3 | ShNF-YA8 | 0.12952 | 0.73661 | -1.45157 | 0.957505 |
| TRINITY_DN226448_c0_g1_i11 | ShNF-YA3 | 0.142148 | 0.750896 | -1.31315 | 0.894612 |
| ShNF-YA3 | ShNF-YA3 | 0.174271 | 0.783444 | -0.94795 | 0.697732 |
| TRINITY_DN218789_c0_g1_i15 | ShNF-YA6 | 0.17707 | 0.786051 | -1.7642 | 1.306975 |
| TRINITY_DN218789_c0_g1_i10 | ShNF-YA6 | 0.241401 | 0.833983 | 1.08 | 0.921901 |
| TRINITY_DN238653_c0_g4_i1 | ShNF-YC2 | 0.243379 | 0.83509 | 1.064894 | 0.912833 |
| TRINITY_DN222879_c1_g5_i3 | ShNF-YA1 | 0.250001 | 0.83746 | -0.76375 | 0.663932 |
| TRINITY_DN222879_c1_g5_i6 | ShNF-YA1 | 0.265398 | 0.84606 | 1.343897 | 1.20667 |
| TRINITY_DN222855_c0_g1_i5 | ShNF-YA5 | 0.27079 | 0.848388 | 1.635604 | 1.485233 |
| ShNF-YB3 | ShNF-YB3 | 0.316037 | 0.867607 | 0.676235 | 0.674458 |
| TRINITY_DN218159_c0_g1_i3 | ShNF-YB3 | 0.329115 | 0.873484 | 1.198365 | 1.227962 |
| TRINITY_DN220412_c0_g2_i2 | ShNF-YC4 | 0.379977 | 0.887727 | 1.183771 | 1.348354 |
| TRINITY_DN222879_c1_g5_i4 | ShNF-YA1 | 0.393635 | 0.890937 | 0.978953 | 1.147599 |
| TRINITY_DN218789_c0_g1_i5 | ShNF-YA6 | 0.399149 | 0.891418 | 1.116315 | 1.323994 |
| TRINITY_DN222879_c1_g5_i2 | ShNF-YA1 | 0.405117 | 0.893027 | -0.21083 | 0.253249 |
| ShNF-YC4 | ShNF-YC4 | 0.424789 | 0.897931 | -0.28622 | 0.358609 |
| TRINITY_DN220412_c0_g2_i1 | ShNF-YC4 | 0.431366 | 0.900104 | 1.082106 | 1.375228 |
| TRINITY_DN233450_c0_g2_i1 | ShNF-YA8 | 0.449797 | 0.903633 | 0.627118 | 0.829791 |
| TRINITY_DN218789_c0_g1_i17 | ShNF-YA6 | 0.501136 | 0.912812 | 0.981857 | 1.459568 |
| TRINITY_DN218789_c0_g1_i9 | ShNF-YA6 | 0.529788 | 0.91897 | -0.75477 | 1.201226 |
| TRINITY_DN230864_c0_g5_i2 | ShNF-YA7 | 0.659262 | 0.945842 | -0.28175 | 0.63899 |
| TRINITY_DN232303_c1_g1_i1 | ShNF-YA4 | 0.674835 | 0.948429 | 0.330923 | 0.788811 |
| TRINITY_DN218789_c0_g1_i8 | ShNF-YA6 | 0.675665 | 0.948535 | -0.59103 | 1.412649 |
| TRINITY_DN218789_c0_g1_i6 | ShNF-YA6 | 0.713154 | 0.956374 | 0.476302 | 1.295623 |
| TRINITY_DN238653_c0_g4_i2 | ShNF-YC2 | 0.724219 | 0.957675 | -0.09365 | 0.265431 |
| ShNF-YA5 | ShNF-YA5 | 0.745722 | 0.961749 | -0.38594 | 1.190126 |
| ShNF-YB2 | ShNF-YB2 | 0.822107 | 0.973245 | -0.0715 | 0.318028 |
| ShNF-YA6 | ShNF-YA6 | 0.839712 | 0.976224 | 0.060569 | 0.299458 |
| ShNF-YA4 | ShNF-YA4 | 0.874554 | 0.98131 | -0.06033 | 0.382122 |
| TRINITY_DN226448_c0_g1_i6 | ShNF-YA3 | 0.893158 | 0.983972 | 0.170973 | 1.272979 |
| TRINITY_DN218789_c0_g1_i14 | ShNF-YA6 | 0.897075 | 0.984798 | -0.23277 | 1.799393 |
| ShNF-YC2 | ShNF-YC2 | 0.937061 | 0.990843 | 0.053006 | 0.671266 |
| TRINITY_DN218789_c0_g1_i13 | ShNF-YA6 | 0.963882 | 0.995246 | 0.070597 | 1.559033 |

## **Dataset S8:** Sequences for *ShNF-Y* genes

## Nucleotide sequences:

>ShNF-YA1 (DN222879)

ATGGAGTCGCGGCCGGGCGGGACGAACCTGGTGGAGCCCAGAGGGCAGGGCGCCGCGCTGCCGTCCGGTGGAGGCCCGGCGGTGCAGCCGTGGTGGACGAGCTCCGGGGCCGTGCTCGGTGCAGTTTCGCCAGCCGTTGTGGCGCCCGGGAGTGGGACGGGGATTAGCCTGTCGAGCAGCCCGGCAGGTGGTAGTGGTGGCGGAGGCGCGGCTAAAGGAGCCCCGAGTGACGAGAGCAGCGAGGATTCACGGAGATCTGGGGAACCAAAAGATGGAAGCGCTGGTCAAGAAAAGAACCATGCCACATCGCAGATACCTGCTTTGGTGCCAGAGTATTTGGCACCATACTCACAGCTGGAACTGAACCAATCAATTGCTTCTGCAGCATATCAGTACCCAGATCCTTACTATGCAGGCATGGTTGCTCCCTATGGAAGTCAAGCTGTGGCTCATTTTCAGTTACCTGGACTAACTCAATCTAGAATGCCATTACCTCTTGAAGTATCCGAGGAGCCTGTTTATGTAAATGCCAAGCAGTACCATGGTATTTTAAGACGACGGCAGTCACGTGCCAAGGCTGAACTTGAGAAAAAGGTGGTCAAAGCCAGAAAGCCATATCTTCATGAGTCTCGTCATCAGCACGCGATGAGGAGGGCAAGAGGAAATGGAGGACGCTTCCTAAACACAAAGAAAAGTGACAATGGTACTCCCAATGGTAAAGCTGAACCTAAGAAAGGAGATGAGAACTCCGAGCGTCTCCATGTCCCCCCTGACTTACTACAGCTACGACAGAACGAGGCTTGA

>ShNF-YA2 (Sh_210J01_contig-1_t000010)

ATGGCTGCCGCGCAACCGGAGCTCCTGCACAGGTACCACCATGGCCGTTTCGAGCTTGGCATTGGGCAATCCATGGTGAGTGTGTTCAACAACAACGCCATTGCTGTTGCTGACCATCAGAGCTATGGCAGCGCTGCGTACTATCCCTTCTACGGAGCCCAAGCTCTGCACGGCGGGAGGGTGCTCCTGCCGCCAGCGATCGCGGCCGAGGAGCCGGTGTACGTGAACGCCAAGCAGTTCAACGGCATCCTCCGGCGGCGCCTGGCGCGCGCCAAGCTTATGGCCGCCAGGGACCGCCGGGTCTCCGGGAACCGCAAGCCATACCTTCACGAGTCACGGCACCTGCACGCGCTGCGCCGGGCGCGGGGCACCGGCGGCCGCTTCCTCAACACCCGGAGCCGCGACGGCGACCCGCACAAGCCGCCGCCGAGGGCAGCGCTGCCAGCACGGAGAAGGCGGCGGCGAGGCTGCAGCAGGATCGGCAGGCGGACGCCTTGTTCCTCTCGTCGCTGGTGA

>ShNF-YA3 (Sh_215C15_t000060)

ATGCCTGTGCTTTTACGGGAAATGGAGGATCATTCTGTCCATCCCATGCCTAAGTCTAACCATGGCTCCTTGTCAGGAAATGGTTATGAGATGAAACATTCAGGCCATAAAGTTTGCGATAGGGATTCAACATCAGAATCTGATCGGCCTCACCAAGAAGCATCAGCAGTGAGTGAGAGCAGTCCAAATGAACACACATCAACTCAATCAGACAATGATGAAGATCATGGGAAGGATCCTCAGGACACAATGAAGCCAGTATTGTCCTTGGGGAAGGAAGGGTCTGCCTTTTTGGCCCCAAAATTAGATTACAGCCCATCTTTTGCTTATATTCCTTATACTGCTGATGCTTATTATGGTGGGGTCTTGACAGGATATGCTCCACATGCCATTGTCCATCCCCAGCAAAATGATACAACAAACACTCCAGTTATGTTGCCTGTGGAACCTGCAGAAGAAGAGCCAATATATGTTAATGCAAAACAATACCATGCAATCCTTAGGAGGAGGCAGACACGTGCTAAATTGGAGGCCCAGAATAAGATGGTGAAAGCTCGGAAGCCATACCTTCATGAGTCTCGACATCGTCATGCCATGAAGCGGGCTCGTGGATCAGGAGGGCGATTCCTCAACACAAAGCAGCTCCAGGAGCAGAGCCAGCAGTATCAGGCATCGAGTGGTTCAATGTGCTCAAAGATCATTGGCAACAGCATAACCTCCCAAAGTGGCCCCACCTGCACGCCCTCTTGTGACACTGCAGGTGCTTCAACAGCCAGCCAGGACCGCAGCTGCTTGCCCTCGGTTGGCTTCCGCCCCACGACGAACTTCAGTCAGCAAGGTGGAGGAGGCTCGAAGCTGGTCGTGAACAGCATGCAGCAGCGTGTTTCCACCATATAA

>ShNF-YA4 (DN_232303)

ATGCTTCTGCGAGAAATGGATGGTGATCCATTCCATCCCATGCCCAACTATGATTTCCTGTCTGGGAATGGTTATTCACTAAAGCAGTTAGTTCGTAGCAACTCTGATATAGACTCTTCATCATCCAAATCTGAGCAGTCTCGGCAAGACTTATCTGACAGCAGCCTCAATGGACAACGCACACCAACACAATCTGATAACAACGATATTTGTGGAAAGCGGGACCAGGGCATGGTGAAGTCTGTATTGTCCTTTGGTAGCCCGGAAGCTGCCTTTTCCCCTCACAAGTTTGACTACAGCCAGTCTTTCGCTTGTGCTTCTTATACTGCTGATCCATATCATGGTGGGGTGTTGGCAGGATACGCTTCAAATGGAATTGTTCATTCCCAAATTAATGGTGCAGCAAACACTAGGGTACCATTGCCTGTTGGGCCTGCAGCAGAGGAACCCATATTTGTCAATGCAAAGCAATACAATGCTATCCTCCGGAGGAGGCAAATACGTGCAAAATTGGAGGCTCAAAATAAACTGGTGAAAGGTCGGAAGCCATATCTCCATGAATCTCGGCATCGTCACGCAATGAAGCGAGTCCGTGGACCAGGAGGCCGTTTCCTCAACAAAAAGGAGCTCCAGGAGCAGCAGCAGAAGGCTCTGCCTTCACTTCAGACTCCAACAGGCGGGGTCAGCAGCAAAATGGCCTTCGGCAGGAACCTATGCACTGAAAGCAGGACATCTCACTCGCCTTCGACGAGCTCTGGGATCTCAAGTGTTTCAAACGGGAGTGGCATGCTGGCTCATCAAGAGCACATCAGCTTCGCATCTGCTAACTTCCTCCCCAGCATGAACTTCCGCGCGGAGAATGGAGGTGAAAAGATGGCCGTCAATGGCGTCCGCCACCACACCCCTGTCGTGAGGTGA

>ShNF-YA5 (Sh_142B14_t000070)

ATGCAGCCGAGGGGGCCAGACCAGAGGAAGGGACGGCAGGGAGGTGGCAAGGCGCAGAGAGCAGCCTTTGCTTGGCGGACGCACCGAGGGAGGCGCGTGGGAGCCATGCTTCTCCCCTCTTCGTCTTCCTCTCCCGCTTCCAAAGGTAACTCCTCCGGAAACACAGTTAATGATCATATGAGATCAACTTTGAGTTTTGATAACAAGCAACATCCATTTGCAAGTCAAAACATTGACTACGGTCAAACAATAGCTTGCATTTCATACCCGTACAATCATTCTGGCTCAGGAGGTGTTTGGGCAGCCTATGAGTCTGGCACCACCGCTGCCACTGTGTTCCATTCCCAAATTTCTGGTGGGGGTACATCCGCAAGAATTCCCTTACCTTTGGAATTAGCAGAGAATGAACCCATATACGTGAATCCCAAACAATATCATGGGATACTTCGCAGAAGACAGTTACGTGCCAAGTTAGAGGCTCAGAACAAGCTAGTCAAAGCCCGAAAGCCTTACCTTCATGAATCTAGGCACCTTCATGCAATGAAGAGGGCACGAGGTTCCGGTGGACGATTCCTCAACACTAAGCAGCTCCAGCAGCAACAGCAATCTCACACTGCCTCCACCAGGTCCACCACAAATGGCACAAGCTCCTCAGGCTCAACTCATCTACGGCTTGGTGGTGGCGCAGCTGGAGATCTATCTATGTTGGCACCCAAAACAATGGCCTCACATGACAGTAGCAAGAAGGCTGTTTCTTCAGCTCTTGCCTTCACTGTGACTCCAATGTTGCGCAGAGATGACGCCTTCTTGCAGCACCCAAGCCACCATCTCAGTTTTTCTGGCCACTTCGGGCAGGCAAGCGCGCAAGCTGGGATGCATAATGGAAGTCAGCATAGGGTTCCAGTTATGAGATGA

>ShNF-YA6 (Sh_239I08_t000130)

ATGAGACATAATGGCACGGTAATGATTCAATTTGGTCATCAAATGCCTGATTACGACTCCCCAGCTACCCAGTCAACCAGTGAGAGCCATCAAGAAGTGTCTGGAATGAGCGAAGGAAGCCTCAACGAGCATAATGATCATTCAGGTAATCTTGATGGTTACTCAAAGAGTGATGAAAACAAGATGATGTCAGCTTTATCCCTGGGTAATCCAGAAACGGGTTATGCACATCCCAAGCCTGACCGTACTCAGTCCTTTGCTATATCATACCCATATGCTGATCCATACTATGGTGGTGCAGTGGCAGCTTATGGCTCACCTGCTATTATGCACCCTCAGCTGGTGGGCATGGTTTCGTCCTCTCGAGTGCCATTACCAATCGAGCCAGCCGCTGAAGAACCCATTTATGTCAACGCGAAACAATACCACGCGATTCTCCGAAGGAGACAGCTCCGTGCAAAGCTAGAGGCTGAAAACAAGCTTGTCAAAAGTCGCAAGCCGTACCTCCACGAGTCTCGGCATCTGCATGCGATGAAGAGAGCCCGGGGAACAGGCGGGCGGTTCCTGAACACGAAGCAGCAGCCGGAGTCACCCGGCAACGGCAGTGGCGGTGGCTCCTCGGATGCGCAACGCATGCCCGCGAACGGCGGCCTGTTCACGAAGCATGAGCACAGCTTGCCACCCGGCGATCGCCACCACTATCACGCGAGAGGGGGCGGCGCGTAG

>ShNF-YA7 (Sh_254O14_t000040)

ATGACCTCTGTTGTTCAGAGTGTTTCAGGTGACCACAGGGCTGAGGATCAACACCATCAGAAGAAGCAAGCTGAACCTGGGGACCAGCAAGAAGCCCCAGTTACTAGTTCAGATAGCCAACCAACAGTGGGCACACCATCAACAGATTATGTGGCACCCTATGCCCCTCATGACATGAGCCATGCAATGGGTCAATACGCTTATCCAAATATTGACCCATACTATGGAAGCCTTTATGCGGCTTACGGTGGACAGCCATTGATGCATCCACCGTTAGTCGGAATGCATCCGGCTGGCTTACCTTTGCCTACCGATGCAATCGAAGAGCCTGTGTATGTAAATGCAAAGCAATACAATGCAATATTAAGACGGCGTCAATCTCGGGCTAAAGCCGAATCAGAACGGAAGCTTGTCAAGGGCCGTAAGCCCTATCTCCATGAGTCACGGCATCAGCATGCCTTGAAAAGGGCCAGGGGAGCTGGAGGTCGATTTCTCAATTCAAAGTCAGATGACAAGGAAGAGAACTCCGACTCAAGTCACAAAGAGAAACAGAATGGAGTTGCGCCCCACAAGAGTGGCCAACCGTCAACCCCTCCGTCTCCCAACGGTGCATCATCAGCTAACCAGGCAGACAGTCGTGAATGA

>ShNF-YA8 (Sh_232I01_t000070)

ATGATGAGCTTCAGGAGCACCCACGAGGGGGCCGGGTTCGGCCACGCCGCCGCCGCAGCCAGCGGAGCGCCGCTGCCGTGGTGGGCAGGGCCGACGGCGGCCCCGATGCTGTGCGGCGAGCCGTTGGGCCTGGGGAGGACGGTGCCGGCCCTGTCGCCGGAGGACCACTGCCGGGATGGCCGCTTCCAGGTCCTCCAAGGACCGCTGGATCCTCCCGTGCCGTCGCTCAAGGCTCCTGTCGCGCAGCAGCAGCCGGACAGGGGGCTCCCGGAACTCCTCAACCTCTCCGTGGCTCATCAAGGTAAAGGGAAGAAAGGTTCTGAGCACTCTGCCACTGTTGCTTTGCAATCACCATTCGCAATATACAATGGTCGTTTTGAGCTGGGCCTTGCTCAGTCAATGATTTCTGCTGATAATTCTTATGCTGACCAACACTATGGCCTGCTTTCTCCTTATCCAATGGGGGCAACGCCTGGAGGGCGTATGCTTATACCACTGAATATGCCAACAGAAGCACCAATTTATGTGAATGCTAAGCAATATGACGCCATTATGCGTCGCCGCCGTGCCCGTGCCAAAGCAGAGAGGGAAAATAGGCTGCTCAAAGCCAGAAAGCCATATCTCCATGAGTCACGCCATCAGCATGCACTGCGTCGCCCACGAGGCTCTGGTGGGCGTTTCCTGAACACAAAGAAAGAATCCAACGGGAAGGATGCTGGTGGAGGCAGAAAGGCAATGTTCAGCAACCCCCTTATGCGCCAGGTGGCTTCTCCAAGTTCAGAGATTCAGCAGTCAGACCTGGGCAACCTGAGCAGCGTCTCCAGCATGTCGGGTTCGGAGGTGTCAAGCATGTATGACCGTGAAGACATGGATCACTACCACAGCTTGGATCACCTCCGCACGCCCTTCTTCACCCCGCTCCCGAGCATCATGGACGGCGAGCATGGGGTCGTTGGTAACCCCTTCAAATGGGCAGCAGCCTCTGAAGTCTGCTGCGACCTCCTGAAAGCATGA

>ShNF-YA13 (Sh_022O20_t000060)

ATGCACCTTTCCTTAACAATGACTCTACAGGCACCAGCGAGCTGGCAACTCCCAGATAGTTTCCATACATGCCAGTGTTTATTCACACATCTTGAGGCATCCGTCCATCATATATACACCTTGTTTATCAGATTTTTCATTATAAAAATACTACTTTTGTGGCCTTTGTCTGATCAGTTATATTTAAAGCAGTTCCATTCCCAAATTTCTGGTGGGGGTACATCCGCAAGAATTCCCTTACCTTTGGAATTAGCAGAGAATGAACCCATATACGTGAATCCCAAACAATATCATGGGATACTTCGCAGAAGACAGTTACGTGCCAAGTTAGAGGCTCAGAACAAGCTAGTCAAAGCCCGAAAGCCTTACCTTCATGAATCTAGGCACCTTCATGCAATGAAGAGGGCACGAGGTTCCGGTGGACGATTCCTCAACACTAAGCAGCTCCAGCAGCAACAGCAATCTCACACTGCCTCCACCAGGTCCACCACAAATGGCACAAGCTCCTCAGGCTCAACTCATCTACGGCTTGGTGGTGGCGCAGCTGGAGATCTATCTATGTTGGCACCCAAAACAATGGCCTCACATGACAGTAGCAAGAAGGCTGTTTCTTCAGCTCTTGCCTTCACTGTGACTCCAATGTTGCGCAGAGATGACGCCTTCTTGCAGCACCCAAGCCACCATCTCAGTTTTTCTGGCCACTTCGGGCAGGCAAGCGCGCAAGCTGGGATGCATAATGGAAGTCAGCATAGGGTTCCAGTTATGAGATGA

>ShNF-YB1 (Sh_241A20_p000070)

ATGGGCCGCAAGGCGAAGCAATGTCCAGCGAAGAAGGGCTGCCGTGACGAGAAGGTCGCCGCGGCGCCGGCAGAGGGCGCGTCGTCGTCGGACGGGGAGGGAGGCACAGGCACCCGCGGGCTGCCGATGGCTAACCTCGTGCGCCTCATGCGGCAGGTAATCCCCAAGCGCGCCAAGATCTCCTCGCGCGCCAAGGAAATTACGCACGACTGCGCCCTCGAGTTCGTCGGCTTCCTCACCGGCGAAGCGGCCGAGCGGGCCACGGCGCAGCACCGCCGCACCATCGCGCCCGAGGACTTCACGTGCTCGCTCCAGGCGCTCGGCTTCGACGACTACGTCAGCCGCCGCACCATCCCGTTATGCTGTGACTCTTTTATTATATCACAAAAATATATAAAGTTATATGTCTTATGA

>ShNF-YB2 (Sh_2441P06_p000030)

ATGGCGGACGCTCCGGCGAGCCCCGGGGGCGGCGGCGGGAGCCACGAGAGCGGGAGCCCCAGGGGCGGCGGAGGTGGAGGCGGTGGCAGCGTCAGGGAGCAGGACAGGTTCCTGCCCATCGCCAACATCAGTCGCATCATGAAGAAGGCCATCCCGGCTAACGGGAAGATCGCCAAGGACGCCAAGGAGACCGTGCAGGAGTGCGTCTCCGAGTTCATCTCCTTCATCACTAGCGAAGCGAGTGACAAGTGCCAGAGGGAGAAGCGGAAGACCATCAACGGCGACGACCTGCTGTGGGCCATGGCCACGTTGGGGTTTGAGGACTACATTGAACCCCTCAAGGTGTACCTGCAGAAGTACCGAGAGATGGAGGGTGATAGTAAGTTAACTGCAAAAACCGGCGATGGCTCAATTAAAAAGGATGCCCTTGGTCATATGGGAGGAAGTACCTCAGCTGCACAAGGGATGGGCCAACAAGGAGCATACAACCAAGGAATGGGTTATATGCAACCTCAGTACCATAACGGGGATATCTCAAACTAA

>ShNF-YB3 (DN_218159)

ATGGCGGACGACGGCGGGAGCCACGAGGGCGGCGGCAGCGGCGGCTACCGGGAGCAGGACCGATTCCTGCCCATCGCCAACATCAGCCGGATCATGAAGAAGGCCGTCCCGGCCAACGGCAAGATCGCTAAGGACGCTAAGGAGACTCTGCAGGAGTGCGTCTCCGAGTTCATATCCTTTGTCACCAGCGAGGCCAGCGACAAATGTCAGAAGGAGAAGAGAAAGACGATCAACGGGGACGATTTGCTTTGGGCGATGGCTACGTTAGGATTCGAGGAGTACGTCGAGCCTCTGAAGATTTACCTACAAAAGTACAGAGAGATGGAGGGTGATAGTAAGCTGTCTACAAAGGCTGGCGAGGGCTCTGTAAAGAAGGATGCAATCAGTTCCCATGGTGGCACCAGTAGCTCAAGTAACCAGTTGGTTCAGCATGGAGTTTACAACCAAGGGATGGGCTATATGCAGCCACAGTACCACAACGGGGATACCTAA

>ShNF-YB4 (Combined Sh_235I12_p000020_213G15)

ATGTCGGAAGCGGAGGCCGCCGGCGGCAGCGGCAGCGGCGGGGGCAAGGAGCAGGACCGCTTCCTGCCGATCGCCAACATCGGGCGCATCATGCGCCGCGCGGTGCCGGAAAACGGCAAGATCGCCAAGGACGCCAAGGAGTCCATCCAGGAGTGCGTCTCCGAGTTCATCAGCTTCATCACCAGCGAAGCGAGTGACAAGTGCATGAAGGAGAGGCGAAAGACCATCAACGGCGACGACATCATCTGGTCCTTGGGCACGCTCGGCTTCGAGGAATACGTTGAGCCTCTCAAGATCTACCTCAAGAATTACCGGGAGACAGAGGGTGACACAAAGGGTTCAAAATCTTCTGATCAGAATGGAAAGAAACAGATTTTACTCAATGGTGAACCTGCATCATCAGTAATATTTACTTTCTGGTTTCAAAACTGCCGTCATATCATTTTTGAATTTGTACAGGGTTTACTTCGCATATTTTGTTTCTAA

>ShNF-YB4 (Sh_243O13_p000150)

ATGAGTGAGAATAATTTCAAGTTCATCGGCTTTGCCCAATTGGGGCTACCCCAACCCCAAAACCTTCATAGGGCATCAAATTCAACTTCATCAGGTAATGCCGACAGCAGCGTTTGCCATGACAATCTCCTACCGATTGCCAACGTTGGGCAGATCATGAAGGACGCCCTCCCACCACAAGCCAAGATATCGAAGCACGCCAAGGAGACCATCCAGGAGTGTGCAATAGAGTTTGTGGGCTTCGTCATCGGCGAGGCCTCCGAGCGGTGCTGTCGGGAGCGGCGGAAGACGATCAACGGCGATGACATCTGTCATGCCATGAGGCACCTTGGCCTGGACCACTACGCTGGAGCCATGCAAAGGTACTTGCAGAGGTATCACGAGAGTGAGGAGCTAGTGGTGGCACTCAACAACAGTGGCATCGGCAGCGGCGATGGCAACAAAGCCATTCAGATCGATGTAAGGGATGAGCTATCCATCTTCAGGGGAAACGAGCAGTAA

>ShNF-YB6 (Sh_241A20_p000080)

ATGGACTCCAGCTTCCTCCCTGCCGGCGGCGCGGACAATGGCTCGGCGGGCGGTGCCAACAACGGCGTCGCTCAGCAGGCGCCGCCGCTGATCCGCGAGCAGGACCGGCTGATGCCAATCGCGAACGTGATCCGCATCATGCGGCGCGTGCTGCCGGCGCACGCCAAGATCTCGGACGACGCCAAGGAGACGATCCAGGAGTGCGTGTCCGAGTACATCAGCTTCATCACGGGGGAGGCCAACGAGCGGTGCCAGCGGGAGCAGCGCAAGACCATCACCGCCGAGGACGTGCTGTGGGCCATGAGCCGCCTCGGCTTCGACGACTACGTCGAGCCGCTCAGCGTCTACCTCCACCGCTACCGCGAGTTCGAGGGCGAGGCGCGGGGCGTCGGGCTCGCCCCGGGGGCCCCTGCGCGCGGCGGCGGCGGCGACCACCACCACCACCCACTCAAGTCCCGCGGGCCCGGCTCCGGAGCCGCCATGTTACCGCACCACCACCACGACATGCAGATGCACGCCGCCATGTACGGGGGCGCCGTGCCCCCGGCGCCGGGGCCTCCACGCCACGGAGGGTTCCTCATGCCACACCCACAGGGCGGCAGCCACTACCTGCCTTACCCCTACGAGCCCACGTATGGCGGCGAGCACGCCATGGCCGCGTACTACGGAGGGGCCGCCGCGTACGCGCCTGGCGACGGCAGCAGCAGCAGCGGCGGGAGAGCGTCGCACACACCGCAGGGCAGCGGCGGCTTCGAGCACCCGCACCCGTTCGCGTACAAGTAG

>ShNF-YB10 (DN_237620)

ATGCCGGACTCCGACAACGAGTCCGGCGGGCCGAGCAACGCGGACTTCTCGTCGCCGCGGGAGCAGGACCGGTTCCTGCCGATCGCGAACGTGAGCCGGATCATGAAGAAGGCGCTCCCGGCCAACGCCAAGATCTCCAAGGACGCCAAGGAGACGGTGCAGGAGTGCGTGTCCGAGTTCATCTCCTTCATCACCGGCGAGGCCTCCGACAAGTGCCAGCGCGAGAAGCGCAAGACCATCAACGGCGACGACCTGCTCTGGGCCATGACCACGCTCGGCTTCGAGGACTACATCGAGCCGCTCAAGCTCTACCTCCACAAGTTCCGCGAGCTCGAGGGCGAGAAGGCGGCCACGGGCGCCGCGGGCTCCTCCTCCGGCGGCTCACAGCCTTAG

>ShNF-YC2 (DN_238653)

ATGGAACCATCCTCTCAGCCTCAGCCTGCAATGGGTGTTGCTGCTGCTGGATCGCAAGTGTATCCTGCCTCTGCCTATCCGCCTGCAGCAACAGTAGCTGCTCCTGCAGTTGCATCTGCTGGTTTACAGTCAGTGCAACCATTCCCAGCCAACCCTGCTCATATGAGTTCTCAGCACCAGATTGTCTACCAACAAGCTCAACAATTCCATCAACAGCTCCAGCAGCAGCAACAGCAGCAGCTTCAGCAGTTCTGGGCTGAACGCATGGCTGAAATTGAGGCGACCACTGATTTCAAGAACCACAACTTGCCACTTGCGAGGATAAAGAAGATCATGAAGGCTGATGAAGACGTTCGCATGATCTCGGCTGAAGCTCCTGTGGTCTTTGCAAAAGCTTGTGAGATATTCATACTGGAGTTGACACTGAGGTCGTGGATGCACACTGAGGAGAACAAGCGCCGCACCTTGCAGAAGAATGACATTGCAGCAGCCATCACTAGGACTGACATTTACGACTTCTTGGTCGACATTGTTCCCAGGGATGAGATGAAGGAGGACGGGGTTGGGCTTCCTAGGGCTGGGTTGCCACCCATGGGAGCCCCAGCTGATGCATATCCATACTACTACATGCAACAGCAGCAGGTGCCTGGTCCTGGAATGGTTTATGGTGCCCAGCAAAGCCACCCAGTGACGTATTTGTGGCAGGAGCCTCAGGAACAGCAGGGGCAAGCTCCTGAAGAGCAGCAGTCTCTGCATGAAAGTGGCTGA

>ShNFYC3 (DN_192942_206940)

ATGTCCAGCAGCAGCTGCACCCTGAAGTGCGTCATGGACACGGAAGGGCAACCCTCTGAGACTGCGGTGAACAAAGGTGTGACAAACAGTGATGCCAATCCTTCAGACTTCCAGGATGCCCCAGTCAACCAACCTCCACTCTCAGATCACCACCAGGTTGCATCCCAGCAAGACGATCACTGCCAGCAACCACTTGAGGCCTTCTGGTCTGGCCAGCTGGCCGAGATCAAGCAGACAGCTAATTTCAAGACCCACAGCCTTCCCCTAGCAAGGATCAAGAAGATCATGAAGGCTGACGCCGACCTTCCTAAAAGGGTTGCGGGCGAGGCACCGTTGCTCTTCGCCAAGGCTTGTGAGATGTTTATCCAGGAATTGACATTGCGGGCGTGGCTTCACACTGAGGAGGACAAGAGGAGGACACTGCAAAAGAAGGATGTCACCGCGGCGCTGGCTAGCACCAAAGTCTTTGATTTCCTGGTGGCCGACGGTTCATCAGACAAGCCAAAGGGGAACGAAGTGGGATTGCCGCCTCCAACAACAACCAACGATGATGATCCCTATGCTGACTACTACAGGGATGACTATTCGCCCCCTTGGTCCCCGGACGAGCCCTCCAGCCCCGACTTGCGCAATGTGACCTGTGATGACCCAAGATACTACACTTACATCCATGATTACTATTCTCTGTGA

>ShNF-YC4 (DN_220412)

ATGGAACCATCCTCACAGCCTCAGCCAGTGATGGGTGTTGGGTCACAACCTTATCCTGCTGCTGCTGCCTATGCACCAACCACAATGGTACCTGGAGCTCCTGCTGTTCCTCCTGGCTCACAGCCAGCAGCACCATTCCCCAACCCAGCTCAACTCAGCGCTCAGCACCAGATGGTCTACCAGCAGGCTCAGCAATTTCACCAACAACTTCAGCAACAGCAGCAACAGCAGCTCAGGGAGTTCTGGACTACCCAGATGGATGAGATCGAGCAAACAACTGACTTCAAGAACCACACCTTGCCACTTGCAAGGATAAAGAAGATAATGAAGGCTGATGAGGATGTGCGGATGATCTCTGCAGAAGCGCCTGTTGTCTTTGCGAAGGCATGCGAGGTGTTCATATTAGAGCTGACATTGAGGTCATGGATGCACACAGAGGAGAACAAGCGCCGGACCTTGCAGAAGAACGACATTGCAGCTGCCATCACCAGGACTGATATATATGACTTCCTGGTGGACATAATCCCGAGGGATGAAATGAAAGAGGAGGGACTCGGGCTTCCAAGAGTTGGCCTGCCGCCTGCCATGGGGGCTCCAGCTGATCATAGCTCTTATCCATATTACTATGTACCAGCACAGCAGGTGCCAGGAGCAGGTATGATGTATGGTGGCCAGCAGCAGGGTCACCCAATGACGTATATGTGGCAGAAGCCGCAAGGGCAAGAGGAAGAGCCTCCCGAAGAGCAGCAGCAGCAGCAGCAGTCTCCTGAGAGTAGCCAGGACATGATAACAGCGAAGCAATTACATCTTGGACCGAACGTAGGCCTCTAA

>ShNFYC6 (Sh_202L17_t000080)

ATGGATCCCAGCAAATCCAGCACCCCTCCGCCGCCTCCAGTCATGGGCGCCCCCGTTGCCTACCCTCCGGCGGCGTACCCTCCCGGCGCGGCCGCCGGCGCCGCCGCCTACGCCCCGCAGCTCTACGCGCCGCCCGCAGCTGCCGCGGTCCAGCAGGCTGCCGCCGCGCAGCAGCAGCAGCTGCAGATGTTCTGGGCGGAGCAGTACCGCGAGATCGAGGCCACCACCGACTTCAAGAACCACAACCTCCCGCTCGCCCGCATCAAGAAGATCATGAAGGCCGACGAGGACGTCCGCATGATCGCCGCCGAGGCCCCCGTCGTGTTCGCCCGGGCCTGCGAGATGTTCATCCTCGAGCTCACCCACCGCGGCTGGGCGCACGCCGAGGAGAACAAGCGCCGCACGCTCCAGAAGTCCGACATTGCCGCTGCCATCGCCCGCACCGAGGTGTTCGACTTCCTCGTGGACATCGTTCCGCGCGACGACGCAAAGGACGCCGACGCGGCGGCCGCCGCAGCTGCTGCGGCTGCCGCTGCCGGGATCCCGCGTCCTGCCGCGGGAGTACCGGCCACCGACCCCCTCGCCTACTACTATGTGCCTCAGCAGTAA

>ShNFYC9 (Sh_201O15_t000030)

ATGGGCGCGCCCGTCGCGTACCCTCCGCCGCCCGGCTCCGCGTACCCCGCCGGGCCGTACGCGCACGCGCCGGCGGCCGCGCTCTACCCTCCTCCCCCGCCGCCGCCGGCTCCCCCCACCTCGCAGCAGGGCGCCGCGGCGGCGCAGCAGCTGCAGCTGTTCTGGGCGGAGCAGTACCGCGAGATCGAGGCCACCACGGACTTCAAGAACCACAACCTGCCGCTGGCCCGCATCAAGAAGATCATGAAGGCCGACGAGGACGTGCGCATGATCGCCGCCGAGGCGCCCGTCGTCTTCGCCCGCGCCTGCGAGATGTTCATCCTCGAGCTCACCCACCGCGGCTGGGCGCACGCCGAGGAGAACAAGCGCCGCACGCTGCAGAAGTCCGACATCGCCGCCGCCGTCGCGCGTACCGAGGTCTTCGACTTCCTCGTCGACATCGTGCCGCGGGACGAGGCCAAGGACGCCGACTCCGCCGCCGCCGCCATGGGAGCGGCCGGGATCCCGCACCCCGCCGCCGACCCCATGGGCTACTACTACGTCCAGCCACAGTAA

## Amino acid sequences:

>ShNF-YA1 (DN_222879)

MESRPGGTNLVEPRGQGAALPSGGGPAVQPWWTSSGAVLGAVSPAVVAPGSGTGISLSSSPAGGSGGGGAAKGAPSDESSEDSRRSGEPKDGSAGQEKNHATSQIPALVPEYLAPYSQLELNQSIASAAYQYPDPYYAGMVAPYGSQAVAHFQLPGLTQSRMPLPLEVSEEPVYVNAKQYHGILRRRQSRAKAELEKKVVKARKPYLHESRHQHAMRRARGNGGRFLNTKKSDNGTPNGKAEPKKGDENSERLHVPPDLLQLRQNEA-

>ShNF-YA2 (Sh_210J01_contig-1_t000010)

MAAAQPELLHRYHHGRFELGIGQSMVSVFNNNAIAVADHQSYGSAAYYPFYGAQALHGGRVLLPPAIAAEEPVYVNAKQFNGILRRRLARAKLMAARDRRVSGNRKPYLHESRHLHALRRARGTGGRFLNTRSRDGDPHKPPPRAALPARRRRRRGCSRIGRRTPCSSRRW-

>ShNF-YA3 (Sh_215C15_t000060)

MPVLLREMEDHSVHPMPKSNHGSLSGNGYEMKHSGHKVCDRDSTSESDRPHQEASAVSESSPNEHTSTQSDNDEDHGKDPQDTMKPVLSLGKEGSAFLAPKLDYSPSFAYIPYTADAYYGGVLTGYAPHAIVHPQQNDTTNTPVMLPVEPAEEEPIYVNAKQYHAILRRRQTRAKLEAQNKMVKARKPYLHESRHRHAMKRARGSGGRFLNTKQLQEQSQQYQASSGSMCSKIIGNSITSQSGPTCTPSCDTAGASTASQDRSCLPSVGFRPTTNFSQQGGGGSKLVVNSMQQRVSTI-

>ShNF-YA4 (DN_232303)

MLLREMDGDPFHPMPNYDFLSGNGYSLKQLVRSNSDIDSSSSKSEQSRQDLSDSSLNGQRTPTQSDNNDICGKRDQGMVKSVLSFGSPEAAFSPHKFDYSQSFACASYTADPYHGGVLAGYASNGIVHSQINGAANTRVPLPVGPAAEEPIFVNAKQYNAILRRRQIRAKLEAQNKLVKGRKPYLHESRHRHAMKRVRGPGGRFLNKKELQEQQQKALPSLQTPTGGVSSKMAFGRNLCTESRTSHSPSTSSGISSVSNGSGMLAHQEHISFASANFLPSMNFRAENGGEKMAVNGVRHHTPVVR-

>ShNF-YA5 (Sh_142B14_t000070)

MQPRGPDQRKGRQGGGKAQRAAFAWRTHRGRRVGAMLLPSSSSSPASKGNSSGNTVNDHMRSTLSFDNKQHPFASQNIDYGQTIACISYPYNHSGSGGVWAAYESGTTAATVFHSQISGGGTSARIPLPLELAENEPIYVNPKQYHGILRRRQLRAKLEAQNKLVKARKPYLHESRHLHAMKRARGSGGRFLNTKQLQQQQQSHTASTRSTTNGTSSSGSTHLRLGGGAAGDLSMLAPKTMASHDSSKKAVSSALAFTVTPMLRRDDAFLQHPSHHLSFSGHFGQASAQAGMHNGSQHRVPVMR-

>ShNF-YA6 (Sh_239I08_t000130)

MRHNGTVMIQFGHQMPDYDSPATQSTSESHQEVSGMSEGSLNEHNDHSGNLDGYSKSDENKMMSALSLGNPETGYAHPKPDRTQSFAISYPYADPYYGGAVAAYGSPAIMHPQLVGMVSSSRVPLPIEPAAEEPIYVNAKQYHAILRRRQLRAKLEAENKLVKSRKPYLHESRHLHAMKRARGTGGRFLNTKQQPESPGNGSGGGSSDAQRMPANGGLFTKHEHSLPPGDRHHYHARGGGA-

>ShNF-YA7 (Sh_254O14_t000040)

MTSVVQSVSGDHRAEDQHHQKKQAEPGDQQEAPVTSSDSQPTVGTPSTDYVAPYAPHDMSHAMGQYAYPNIDPYYGSLYAAYGGQPLMHPPLVGMHPAGLPLPTDAIEEPVYVNAKQYNAILRRRQSRAKAESERKLVKGRKPYLHESRHQHALKRARGAGGRFLNSKSDDKEENSDSSHKEKQNGVAPHKSGQPSTPPSPNGASSANQADSRE-

>ShNF-YA10 (Sh_232I01_t000070)

MMSFRSTHEGAGFGHAAAAASGAPLPWWAGPTAAPMLCGEPLGLGRTVPALSPEDHCRDGRFQVLQGPLDPPVPSLKAPVAQQQPDRGLPELLNLSVAHQGKGKKGSEHSATVALQSPFAIYNGRFELGLAQSMISADNSYADQHYGLLSPYPMGATPGGRMLIPLNMPTEAPIYVNAKQYDAIMRRRRARAKAERENRLLKARKPYLHESRHQHALRRPRGSGGRFLNTKKESNGKDAGGGRKAMFSNPLMRQVASPSSEIQQSDLGNLSSVSSMSGSEVSSMYDREDMDHYHSLDHLRTPFFTPLPSIMDGEHGVVGNPFKWAAASEVCCDLLKA-

>ShNF-YA13 (Sh_022O20_t000060)

MHLSLTMTLQAPASWQLPDSFHTCQCLFTHLEASVHHIYTLFIRFFIIKILLLWPLSDQLYLKQFHSQISGGGTSARIPLPLELAENEPIYVNPKQYHGILRRRQLRAKLEAQNKLVKARKPYLHESRHLHAMKRARGSGGRFLNTKQLQQQQQSHTASTRSTTNGTSSSGSTHLRLGGGAAGDLSMLAPKTMASHDSSKKAVSSALAFTVTPMLRRDDAFLQHPSHHLSFSGHFGQASAQAGMHNGSQHRVPVMR-

>ShNF-YB1 (Sh_241A20_p000070)

MGRKAKQCPAKKGCRDEKVAAAPAEGASSSDGEGGTGTRGLPMANLVRLMRQVIPKRAKISSRAKEITHDCALEFVGFLTGEAAERATAQHRRTIAPEDFTCSLQALGFDDYVSRRTIPLCCDSFIISQKYIKLYVL-

>ShF-YB2 (Sh_2441P06_p000030)

MADAPASPGGGGGSHESGSPRGGGGGGGGSVREQDRFLPIANISRIMKKAIPANGKIAKDAKETVQECVSEFISFITSEASDKCQREKRKTINGDDLLWAMATLGFEDYIEPLKVYLQKYREMEGDSKLTAKTGDGSIKKDALGHMGGSTSAAQGMGQQGAYNQGMGYMQPQYHNGDISN-

>ShNF-YB3 (DN_218159)

MADDGGSHEGGGSGGYREQDRFLPIANISRIMKKAVPANGKIAKDAKETLQECVSEFISFVTSEASDKCQKEKRKTINGDDLLWAMATLGFEEYVEPLKIYLQKYREMEGDSKLSTKAGEGSVKKDAISSHGGTSSSSNQLVQHGVYNQGMGYMQPQYHNGDT-

>ShNF-YB4 (Combined Sh_235I12_p000020_213G15p0000030)

MSEAEAAGGSGSGGGKEQDRFLPIANIGRIMRRAVPENGKIAKDAKESIQECVSEFISFITSEASDKCMKERRKTINGDDIIWSLGTLGFEEYVEPLKIYLKNYRETEGDTKGSKSSDQNGKKQILLNGEPASSVIFTFWFQNCRHIIFEFVQGLLRIFCF-

>ShNFYB5 (Sh_243O13_p000150)

MSENNFKFIGFAQLGLPQPQNLHRASNSTSSGNADSSVCHDNLLPIANVGQIMKDALPPQAKISKHAKETIQECAIEFVGFVIGEASERCCRERRKTINGDDICHAMRHLGLDHYAGAMQRYLQRYHESEELVVALNNSGIGSGDGNKAIQIDVRDELSIFRGNEQ-

>ShNFYB6 (Sh_241A20_p000080)

MDSSFLPAGGADNGSAGGANNGVAQQAPPLIREQDRLMPIANVIRIMRRVLPAHAKISDDAKETIQECVSEYISFITGEANERCQREQRKTITAEDVLWAMSRLGFDDYVEPLSVYLHRYREFEGEARGVGLAPGAPARGGGGDHHHHPLKSRGPGSGAAMLPHHHHDMQMHAAMYGGAVPPAPGPPRHGGFLMPHPQGGSHYLPYPYEPTYGGEHAMAAYYGGAAAYAPGDGSSSSGGRASHTPQGSGGFEHPHPFAYK-

>ShNF-YB10 (DN_237620)

MPDSDNESGGPSNADFSSPREQDRFLPIANVSRIMKKALPANAKISKDAKETVQECVSEFISFITGEASDKCQREKRKTINGDDLLWAMTTLGFEDYIEPLKLYLHKFRELEGEKAATGAAGSSSGGSQP-

>ShNF-YC2 (DN238653)

MEPSSQPQPAMGVAAAGSQVYPASAYPPAATVAAPAVASAGLQSVQPFPANPAHMSSQHQIVYQQAQQFHQQLQQQQQQQLQQFWAERMAEIEATTDFKNHNLPLARIKKIMKADEDVRMISAEAPVVFAKACEIFILELTLRSWMHTEENKRRTLQKNDIAAAITRTDIYDFLVDIVPRDEMKEDGVGLPRAGLPPMGAPADAYPYYYMQQQQVPGPGMVYGAQQSHPVTYLWQEPQEQQGQAPEEQQSLHESG-

>ShNF-YC3 (DN_192942_206940)

MSSSSCTLKCVMDTEGQPSETAVNKGVTNSDANPSDFQDAPVNQPPLSDHHQVASQQDDHCQQPLEAFWSGQLAEIKQTANFKTHSLPLARIKKIMKADADLPKRVAGEAPLLFAKACEMFIQELTLRAWLHTEEDKRRTLQKKDVTAALASTKVFDFLVADGSSDKPKGNEVGLPPPTTTNDDDPYADYYRDDYSPPWSPDEPSSPDLRNVTCDDPRYYTYIHDYYSL-

>ShNF-YC4 (DN_220412)

MEPSSQPQPVMGVGSQPYPAAAAYAPTTMVPGAPAVPPGSQPAAPFPNPAQLSAQHQMVYQQAQQFHQQLQQQQQQQLREFWTTQMDEIEQTTDFKNHTLPLARIKKIMKADEDVRMISAEAPVVFAKACEVFILELTLRSWMHTEENKRRTLQKNDIAAAITRTDIYDFLVDIIPRDEMKEEGLGLPRVGLPPAMGAPADHSSYPYYYVPAQQVPGAGMMYGGQQQGHPMTYMWQKPQGQEEEPPEEQQQQQQSPESSQDMITAKQLHLGPNVGL-

>ShNFYC6 (Sh_202L17_t000080)

MDPSKSSTPPPPPVMGAPVAYPPAAYPPGAAAGAAAYAPQLYAPPAAAAVQQAAAAQQQQLQMFWAEQYREIEATTDFKNHNLPLARIKKIMKADEDVRMIAAEAPVVFARACEMFILELTHRGWAHAEENKRRTLQKSDIAAAIARTEVFDFLVDIVPRDDAKDADAAAAAAAAAAAAGIPRPAAGVPATDPLAYYYVPQQ-

>ShNFYC9 (Sh_201O15_t000030)

MGAPVAYPPPPGSAYPAGPYAHAPAAALYPPPPPPPAPPTSQQGAAAAQQLQLFWAEQYREIEATTDFKNHNLPLARIKKIMKADEDVRMIAAEAPVVFARACEMFILELTHRGWAHAEENKRRTLQKSDIAAAVARTEVFDFLVDIVPRDEAKDADSAAAAMGAAGIPHPAADPMGYYYVQPQ-

**a)**


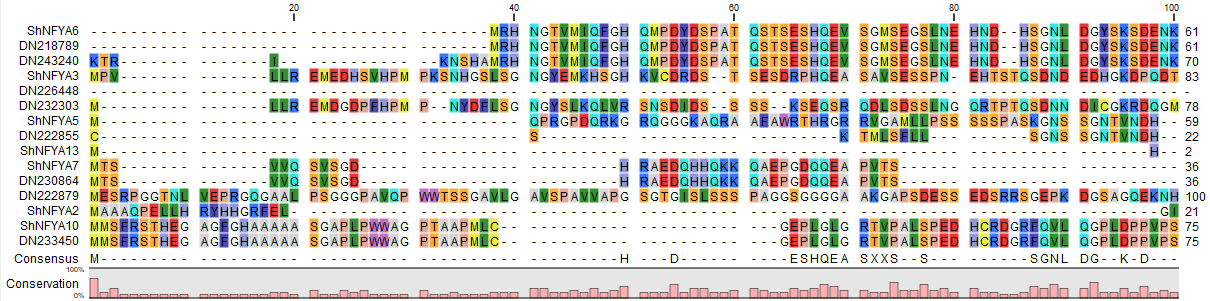


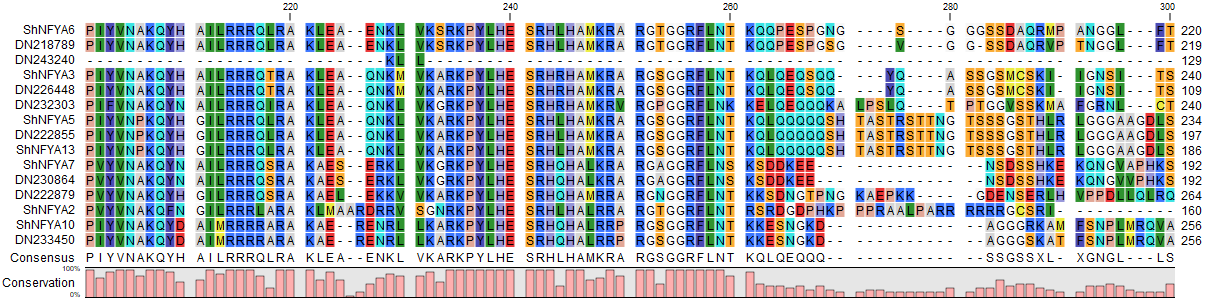


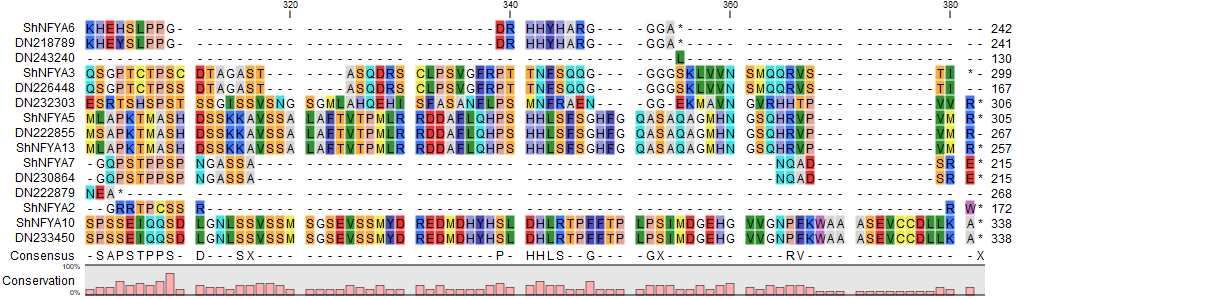


**b)**

**
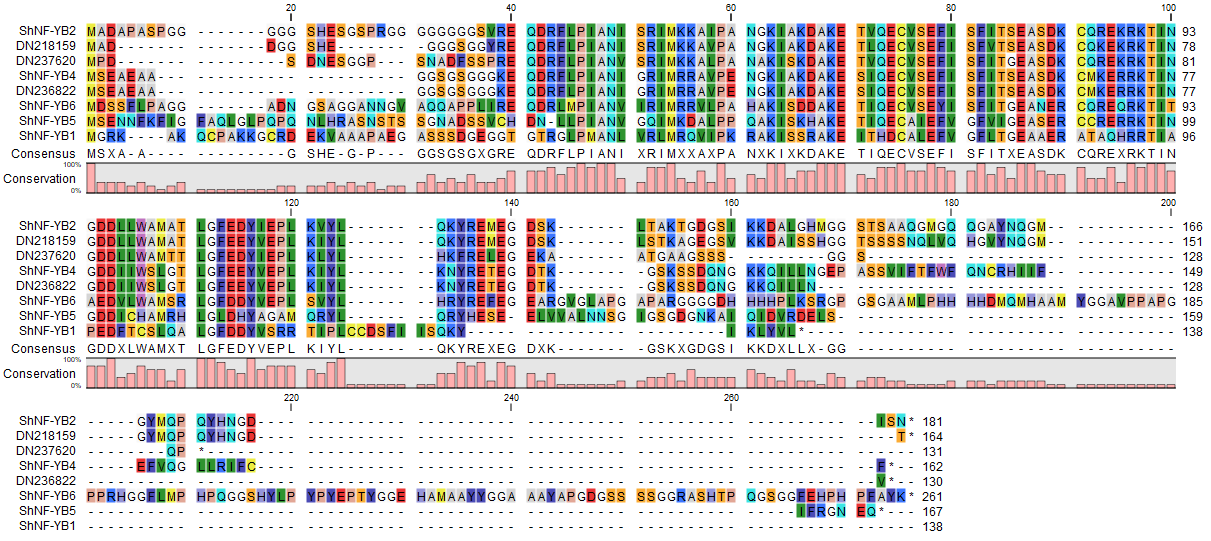
**

**c)**
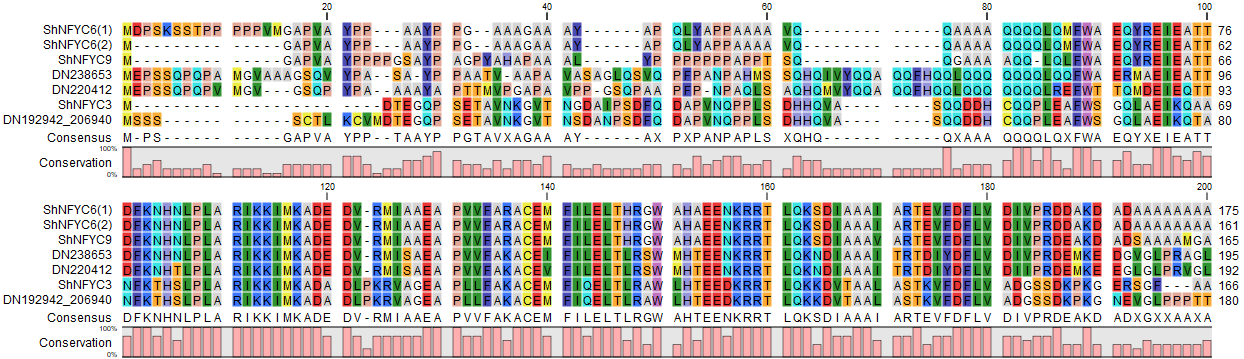


####
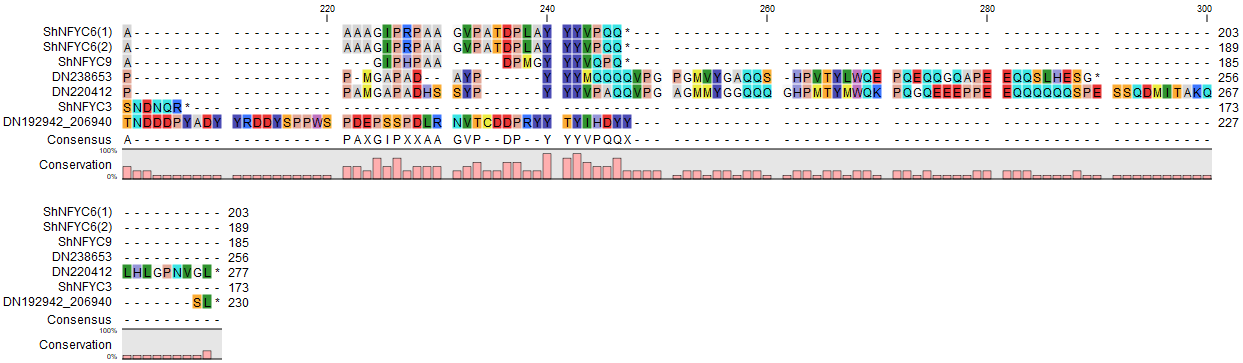


#### **Figure S8:** Multiple sequence alignments of sugarcane amino acid sequences for **a**) NF-YA, **b**) NF-YB and **c**) NF-YC.
